# Emergence of specialized third-party enforcement

**DOI:** 10.1073/pnas.2207029120

**Published:** 2023-06-06

**Authors:** Erik Mohlin, Alexandros Rigos, Simon Weidenholzer

**Affiliations:** ^a^Department of Economics, Lund University, 220 07 Lund, Sweden; ^b^Institute for Futures Studies, 101 31 Stockholm, Sweden; ^c^Department of Economics, University of Essex, Colchester CO4 3SQ, United Kingdom

**Keywords:** evolution of cooperation, evolution of institutions, specialized reciprocity, third-party punishment, policing

## Abstract

The question of how cooperation evolves and is maintained among nonkin is central to the biological, social, and behavioral sciences. Previous research has focused on explaining how cooperation in social dilemmas can be maintained by direct and indirect reciprocity among the participants of the social dilemma. However, in complex human societies, both modern and ancient, cooperation is frequently maintained by means of specialized third-party enforcement. We provide an evolutionary-game-theoretic model that explains how specialized third-party enforcement of cooperation (specialized reciprocity) can emerge. A population consists of producers and enforcers. First, producers engage in a joint undertaking represented by a prisoner’s dilemma. They are paired randomly and receive no information about their partner’s history, which precludes direct and indirect reciprocity. Then, enforcers tax producers and may punish their clients. Finally, the enforcers are randomly paired and may try to grab resources from each other. In order to sustain producer cooperation, enforcers must punish defecting producers, but punishing is costly to enforcers. We show that the threat of potential intraenforcer conflict can incentivize enforcers to engage in costly punishment of producers, provided they are sufficiently informed to maintain a reputation system. That is, the “guards” are guarded by the guards themselves. We demonstrate the key mechanisms analytically and corroborate our results with numerical simulations.

## Significance

In a social dilemma, total resources are maximized if everyone cooperates, but everyone is tempted to not cooperate. Cooperation may be impossible if the parties interact infrequently or do not have sufficient information about each other’s past behavior. In such cases, human cooperation is often maintained by third parties who specialize in enforcement (e.g., a police force). However, if enforcers are powerful enough to punish noncooperators, they may also be powerful enough to extract resources from their clients without providing any services in return. We show that by using a reputation system, enforcers can police each other and sanction those who fail to punish noncooperation in the social dilemma.

Human societies are characterized by high levels of cooperation among large numbers of genetically distant individuals (1). To maintain cooperation, socially complex societies typically rely on third-party enforcement (of, for example, social norms, laws, contracts, and informal agreements), which is often carried out by specialized enforcers (2). While specialized enforcement can contribute to the effectiveness and impartiality of governance institutions —which are fundamental for economic growth and social development (3–5)—, achieving and maintaining socially beneficial institutions of third-party enforcement is not straightforward. Effective enforcement requires powerful enforcers, but if an enforcer is powerful enough to protect her clients from crime, then she may also be powerful enough to extract resources from her clients without providing any services in return.^*^ In the words of the Roman satirist Juvenal, one is led to ask *“who guards the guards?”* In this paper, we develop an evolutionary-game-theoretic model of a system where cooperation is enforced by guards who guard themselves.

Previous literature has investigated different sanctioning mechanisms that enable the evolution and maintenance of cooperation. If the interacting parties face each other repeatedly, cooperation may evolve via direct reciprocity (6) and be sustained in equilibrium as described by the folk theorems of game theory (7–9), reflecting a social institution sometimes referred to as mutual enforcement (10). The importance of punishment as a proximate mechanism promoting cooperation, even in finitely repeated games, has been documented experimentally (11, 12). If there is a sufficiently high probability of interacting with another partner from the same community, and if there is sufficient information about the partner’s past behavior, then cooperation may evolve as described by theories of indirect reciprocity (13–17) and theories of community enforcement (18–23). If the probability of interacting with another partner from the community is too low, and if the information about past behavior is not of sufficient quality, then neither direct nor indirect reciprocity are able to foster cooperation. In this case, the possibility of specialized third-party enforcement becomes crucial for cooperation.

We consider a population where each agent specializes in either production or enforcement. Producers specialize in generating resources for consumption and reproduction. Enforcers are not involved in production but instead specialize in information acquisition and the use of violence and other means of coercion (24). They can coercively tax their producer-clients, identify defectors and cooperators among them (10, 25), and decide whether to punish each of them. In our theory, specialized enforcers employ a reputation system, which allows an enforcer to condition her behavior toward her peers on the latter’s good past behavior. Good enforcer behavior entails punishing defecting clients and refraining from attacking other enforcers, unless they have misbehaved. In this sense, we propose a theory of specialized reciprocity.

We aim to provide an explanation of how third-party sanctioning institutions can emerge and be maintained. It is, therefore, not sufficient to assume that the institutions act in certain ways without deriving their behavior from that of the individuals they comprise (26, 27). Our game-theoretic model takes individuals’ incentives into account and explains why some choose to work for these institutions and to implement costly sanctions. The long-run reputation incentives of enforcers create short-run incentives for producers to cooperate. Thus, we move beyond important recent models of so-called pool punishment (28–30) [c.f. (31)]. Importantly, we do not assume that any enforcer has a local monopoly of violence, neither do we assume that enforcers act as a unitary organization. Instead, a cohesive enforcement organization with a monopoly of violence emerges endogenously in one of the equilibria of the model (32).

## Results

### The Repeated Enforcement Game.

**Fig. 1.**
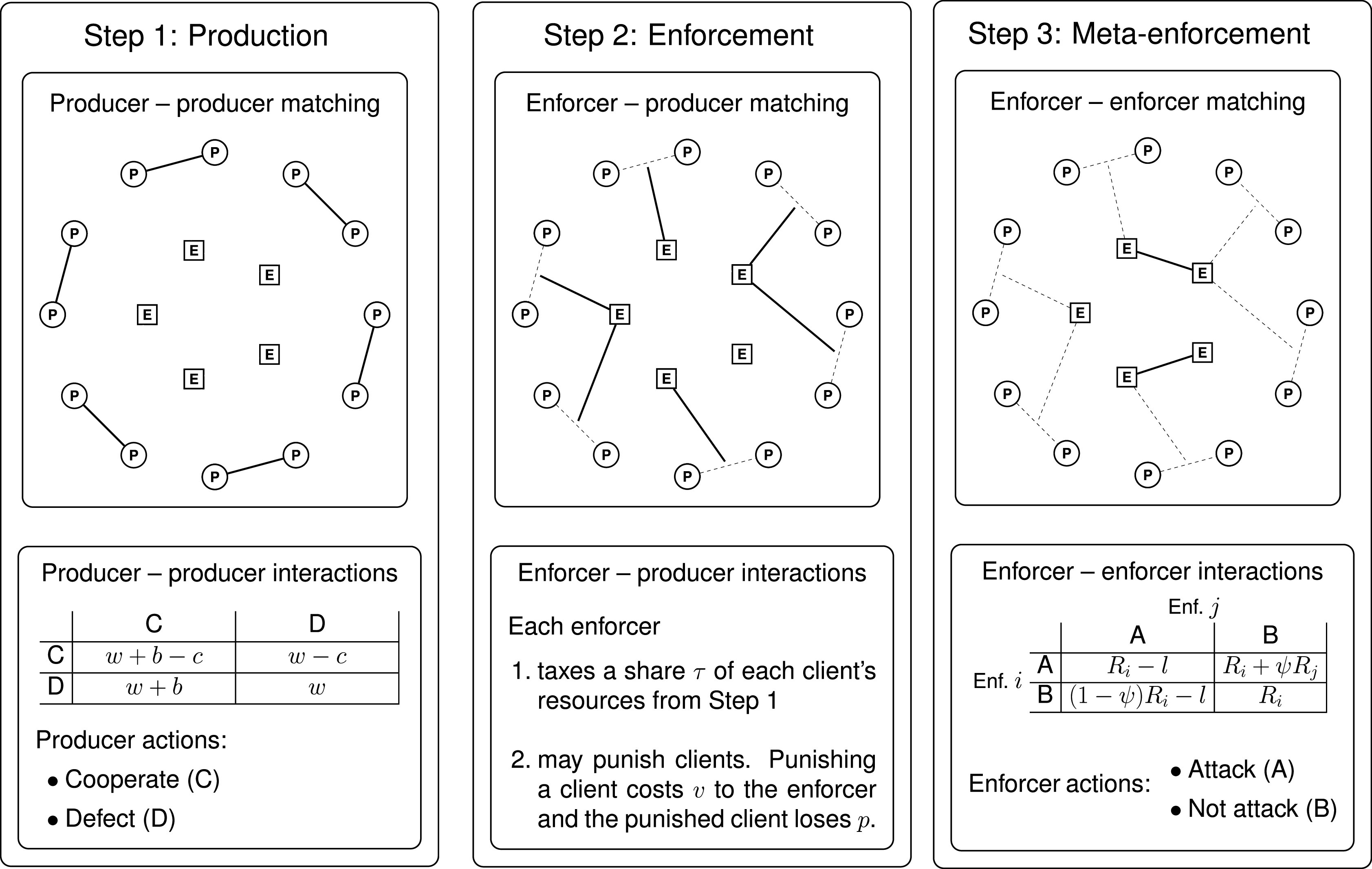
The three steps of the stage game. In the production step (step 1), the producers are randomly matched into pairs to play a PD, choosing to cooperate or to defect. The entries of the payoff matrix represent payoffs to the row player. In the enforcement step (step 2), each producer pair from step 1 is randomly matched to one enforcer. Enforcers tax the producers matched to them and have the opportunity to punish them. In the meta-enforcement step (step 3), the enforcers are matched into pairs. Each can choose to be peaceful or to attack the other over the revenue they took from the producers in the previous step. The entries of the payoff matrix represent payoffs to the row player (*i*) against the column player (*j*), where *R*_*i*_ and *R*_*j*_ represent revenue earned in the enforcement step.

In each time period, the members of the population interact in a repeated game consisting of an indefinite number of rounds. After each round of play, a new round begins with probability *δ*, while the game ends with probability 1 − *δ*. In each round, the agents face a stage game that consists of three steps (Fig. 1). The payoff from the repeated game played in a time period is evaluated as the per-round average payoff.

In the production step, producers are randomly paired. Each pair plays a prisoner’s dilemma (PD), which represents a potentially mutually beneficial interaction (e.g., trade or joint production). Each producer chooses between: i) cooperation, which bestows a benefit *b* upon the partner at a cost *c* < *b* to the cooperator, and ii) defection, which creates no benefit and carries no cost. Producers have autarky payoff *w*, which represents production outside the match.

In the enforcement step, each producer pair is randomly matched to an enforcer, becoming the enforcer’s clients. The enforcer taxes her clients, taking a fraction *τ* of the resources that each accumulated in the production step. We interpret this as a situation where the remaining fraction 1 − *τ* is impossible to transfer (e.g., because it is directly consumed) or can be defended by the producer. The enforcer can also choose to punish any of her clients, inflicting a cost *p* on each. Punishing costs the enforcer *v* per punished producer. We refer to *v* as variable cost because it varies with the number of punished producers. Since enforcers are specialized, the producers are unable to counterpunish.

In the meta-enforcement step, enforcers are randomly paired. Each enforcer *i* chooses to attack (action A) or not attack (action B) her coplayer *j* in an attempt to grab tax revenue (*R*_*j*_) that *j* obtained in the enforcement step. If neither attacks, they both walk away with their respective tax revenues. If one enforcer attacks and the other does not, the attacker takes a fraction *ψ* from the nonattacker, who also suffers a loss *l* from being attacked. If both attack, each suffers a loss *l*, but no revenue changes hands.

### Information, Reputation, and the Cooperation Enforcer Strategy.

We assume that the population is large so that the probability of meeting the same agent soon again is small. Moreover, a producer has no information about the behavior of other producers, apart from knowing what occurred in the interactions she was herself involved in. These assumptions imply that both direct and indirect reciprocity among producers are ineffective.

By contrast, at the beginning of a period, each enforcer can choose to acquire information by paying a fixed cost *f*, which represents the cost and effort of building and maintaining the requisite information channels. If she chooses to do so, then in each round, she obtains two kinds of information: i) information about the outcome of all interactions in the current round in which any of her clients were involved in the production step and ii) information encoded in an enforcer reputation system.

Specifically, the cooperation enforcer (CE) reputation system is based on a CE standard of correct behavior which requires an enforcer to i) punish every act of defection and no act of cooperation among her clients in the enforcement step and ii) attack her coplayer in the meta-enforcement step if and only if that coplayer has bad reputation. Correspondingly, we define the CE strategy which consists in always acting in accordance with the CE standard.

**Fig. 2.**
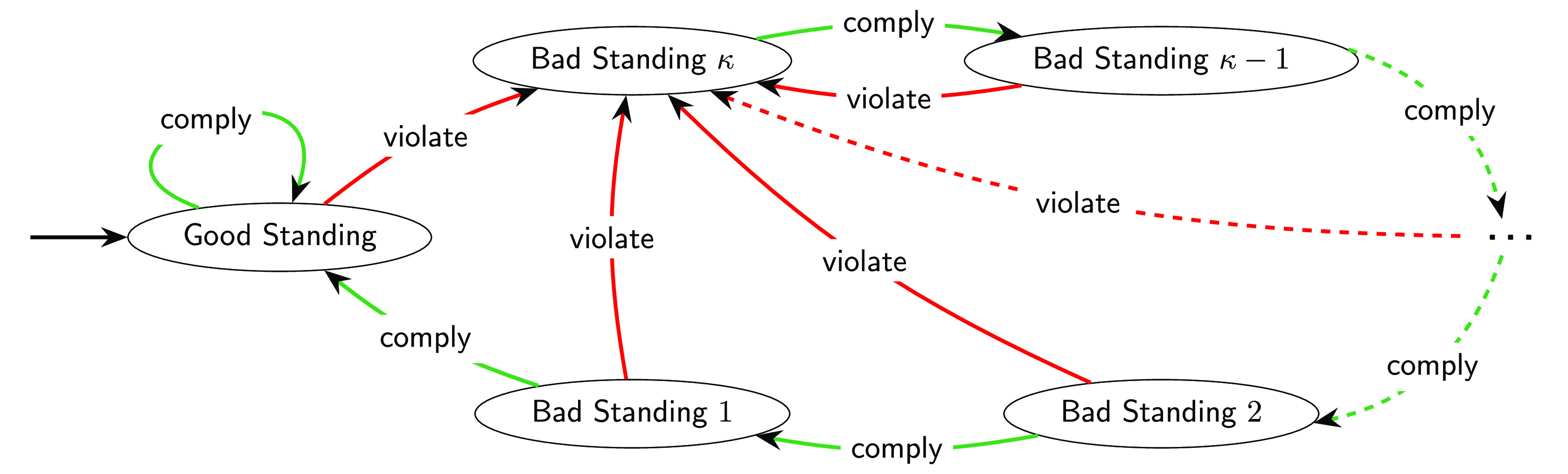
Reputation system among enforcers. All enforcers start out in good standing and remain in good standing as long as they comply with the standard. If an enforcer in good standing violates the standard, she enters bad standing of degree *κ*. For every round that an enforcer in bad standing complies with the standard, she has her degree of bad standing reduced by one. If an enforcer in bad standing violates the standard, she goes back to bad standing of degree *κ*. An enforcer in bad standing who complies with the standard will eventually arrive at a bad standing of degree zero. This means that she is back in good standing. Thus, *κ* is the number of rounds of punishment.

The reputation system classifies agents to be in good standing or in bad standing. If an enforcer is in good standing and complies with the CE standard, she remains in good standing. If an enforcer does not comply with the CE standard, she enters bad standing and remains there for *κ* rounds (during which she can expect to be attacked). If she fails to comply while in bad standing, the punishment phase restarts. See Fig. 2 for an illustration.

Our reputation system is an extension of Kandori’s reputation system (20) adapted to our stage game. In the special case of a PD and a single round of punishment (*κ* = 1), Kandori’s system reduces to the reputation system known as “strict standing” (16) or “stern judging” (33) in the literature on indirect reciprocity. It is one of the “leading eight” reputation systems that have been identified as particularly conducive to cooperation (33–36).

### Static Equilibrium.

As a first test of the viability of maintaining cooperation by means of the CE strategy, we perform a static (nonevolutionary) analysis of the repeated enforcement game employing the concept of subgame perfect Nash equilibrium (37). We use a maximally inclusive set of strategies for the enforcers when checking for possible deviations. For a formal exposition, see *SI Appendix*, S1.

Our analysis identifies two conditions that need to be satisfied. First, the cost of enforcer conflict needs to be high enough relative to both the fixed cost of being an informed enforcer and the variable cost of punishing clients:

[1] $$\begin{array}{c} l>2\;\max\{f,v\}. \end{array}$$

Second, producers’ loss from being punished needs to be high enough relative to the cost of cooperation.

[2] $$\begin{array}{c} p>c\left(1-\tau \right). \end{array}$$

If Eqs. **1** and **2** hold, then, for a long-enough punishment phase (high *κ*) and high-enough repetition probability (*δ*), there is a subgame perfect Nash equilibrium in which all enforcers follow the CE strategy and all producers always cooperate.

As is typical in repeated games, there is a vast number of equilibria with varying levels of cooperation (8, 9). While the full-cooperation equilibrium outcome requires certain conditions to be satisfied, there is always a full-defection equilibrium in which enforcers never punish and always attack.

### Dynamic Framework.

Our static analysis identifies a key mechanism by which enforcers can be incentivized to enforce cooperation among producers, but it cannot reveal which equilibrium (if any) is likely to emerge from a decentralized evolutionary process. To shed light on this question, we embed the repeated enforcement game in a dynamic evolutionary-game-theoretic setting. In addition, our dynamic model has a richer structure than the static model, including the possibility of mistakes and the endogenous determination of the fraction of enforcers.

Between two consecutive time periods, (i.e., after each instance of the repeated game), one agent is drawn to revise her strategy. With probability *γ*_*P*_, the agent is drawn among those currently using producer strategies and can only choose among the producer strategies, *S*^*P*^. With probability *γ*_*E*_, the agent is drawn among those using enforcer strategies and can only choose among the enforcer strategies, *S*^*E*^. With probability *γ*_*P*, *E*_ = 1 − *γ*_*P*_ − *γ*_*E*_, the agent is drawn from the whole population and gets to choose among all (producer and enforcer) strategies, *S*^*P*^ ∪ *S*^*E*^. We assume *γ*_*P*_ > *γ*_*E*_ > *γ*_*P*, *E*_, capturing the idea that revisions among producers are easier than revisions among enforcers, which in turn are easier than revisions across producer/enforcer roles.

When revising, agents tend to select strategies that are currently performing well. With probability 1 − *ε*, a revising agent chooses a noisy (logit) best response (38) with imprecision parameter *η* (such that *η* → 0 corresponds to the exact best response and *η* → ∞ to uniformly random choice). With probability *ε*, the revising agent chooses a strategy at random. We refer to these as revision mistakes (corresponding to mutations in genetic evolution), but they could also be interpreted as acts of experimentation. The resulting dynamic captures boundedly rational social learning or social evolution, rather than genetic evolution (39).

The dynamic constitutes a Markov chain on the set of population states. Since the agents make mistakes that can lead them to adopt any strategy, the process is ergodic. Hence, it induces a unique invariant distribution over the set of strategies (40–44). In the context of imitation-based processes or birth–death processes, this distribution is sometimes referred to as a “mutation–selection equilibrium” (45).

### Strategies.

The full set of strategies is infinite as strategies can be arbitrarily complex (since enforcers who acquire information can condition their actions on each of the infinite possible histories). This was not an obstacle to our static equilibrium analysis. However, a systematic dynamic analysis is feasible only if we restrict attention to a smaller subset of strategies.

Producers use one of two strategies: cooperating producer (CP), who always cooperates, and defecting producer (DP), who always defects. Given the limited information on which producers can condition their action, this is a relatively modest simplification.

Enforcers are restricted to strategies whose action in the enforcement step can be conditioned only on whether the client cooperated or defected in the current round and whose action in the meta-enforcement step can be conditioned only on whether the matched enforcer is in good or bad standing. We assume that strategies that do not condition actions (on either producer behavior or enforcer reputation) do not acquire information and, therefore, do not pay the fixed cost *f*. Since there are two actions available in each of the enforcement and meta-enforcement steps, this defines 2^4^ = 16 enforcer strategies.

We represent each such strategy as a string of four binary digits (zeroes and ones), which respectively correspond to i) the action against a producer who has cooperated, ii) the action against a producer who has defected, iii) the action against an enforcer in good standing, and iv) the action against an enforcer in bad standing. We use 1 to indicate punishment in the enforcement step and attack in the meta-enforcement step. For example, 0101 represents the CE strategy. Another strategy that will play an important role is the defection enforcer (DE) strategy which never punishes and always attacks. It is represented as 0011. Notice that our strategy set includes antisocial punishers (e.g., 1010).

Strategies are not necessarily executed perfectly. Each time an agent faces a choice between actions, with probability 1 − *μ*, the agent takes the action prescribed by her strategy and with probability *μ* she takes the other action by mistake.

We denote the number of agents using a strategy *x* by *n*_*x*_ (e.g., *n*_*CE*_ is the number of CE in the population). Moreover, the total number of producers is *n*_*P*_, and the total number of enforcers is *n*_*E*_. The total population size is *N* = *n*_*P*_ + *n*_*E*_.

### Dynamic Analytical Results.

In order to obtain analytical results regarding the invariant distribution, we need to make a number of simplifying assumptions. As a first simplification, we only consider the CE and DE strategies for the enforcers, in addition to the CP and DP strategies for the producers. Second, we abstract away from action mistakes and variable cost (by setting *μ* = *v* = 0) and consider exact best reply, *η* = 0. Third, we focus on the limit where the (expected) number of rounds per period and the number of punishment rounds get arbitrarily large (*δ* → 1 and *κ* → ∞). Fourth, we assume that punishment is sufficiently severe (*p* > (1−*τ*) *c*) and sufficiently cheap (*f* < *l*). Finally, we let revisions among the producer strategies be arbitrarily more common than revisions among the enforcer strategies, which in turn are arbitrarily more common than revisions among all the strategies. See *Methods* for details.

We first consider the dynamic that arises in the absence of revision mistakes (mutations), i.e., if *ε* = 0. The resulting “unperturbed” best-response dynamic has two absorbing sets of states (see *SI Appendix*, Fig. S1 for an illustration). One of these sets corresponds to a cooperation equilibrium in which only CP and CE are present, while the other absorbing set corresponds to a defection equilibrium in which only DP and DE are present. In the cooperation equilibrium, the fraction of enforcers (CE) is

[3] $$\begin{array}{c} \alpha^{C}=\frac{\tau (w+b-c)}{w+b+f-c}, \end{array}$$

and the fraction of producers (CP) is 1 − *α*^*C*^. In the defection equilibrium, the fraction of enforcers (DE) is

[4] $$\begin{array}{c} \alpha^{D}=\frac{w\tau }{w+l}, \end{array}$$

and the fraction of producers (DP) is 1 − *α*^*D*^. Both equilibria are locally stable. The boundary between the basins of attraction is given by a region that converges to the plane $\frac{n_{\mathrm{CE}}}{n_{E}}=\frac{f}{l}$ in the limit as *N* → ∞. Depending on the initial value of $\frac{n_{\mathrm{CE}}}{n_{E}}$, the process converges to one of the two equilibria (see *SI Appendix*, Fig. S3 for an illustration). It can be verified that payoffs and the share of enforcers are higher in the cooperation equilibrium than in the defection equilibrium (see *SI Appendix*, Observation S1).

Now, consider the dynamic with rare mutations (where *ε* is vanishingly small, but positive). As *ε* → 0, the invariant distribution puts almost all weight on a single state (one of the equilibria). Such a state is called stochastically stable (40–43). For a comparison with other stochastic evolutionary approaches, see *SI Appendix*, S3. When the population is sufficiently large, the cooperation equilibrium is stochastically stable if

[5] $$\begin{array}{c} \left(\frac{l}{f}-1\right)\left(1+\frac{l}{w}\right)>1+\frac{f}{w+b-c}. \end{array}$$

If the inequality is reversed, then the defection equilibrium is stochastically stable. Thus, stochastic stability of cooperation is enabled by a high destructiveness of conflict between enforcers *l*, a low (fixed) cost of information *f*, a high benefit of cooperation *b*, and a low cost of cooperation *c*. The effect of *w* is ambiguous.

### Dynamic Numerical Results.

**Fig. 3.**
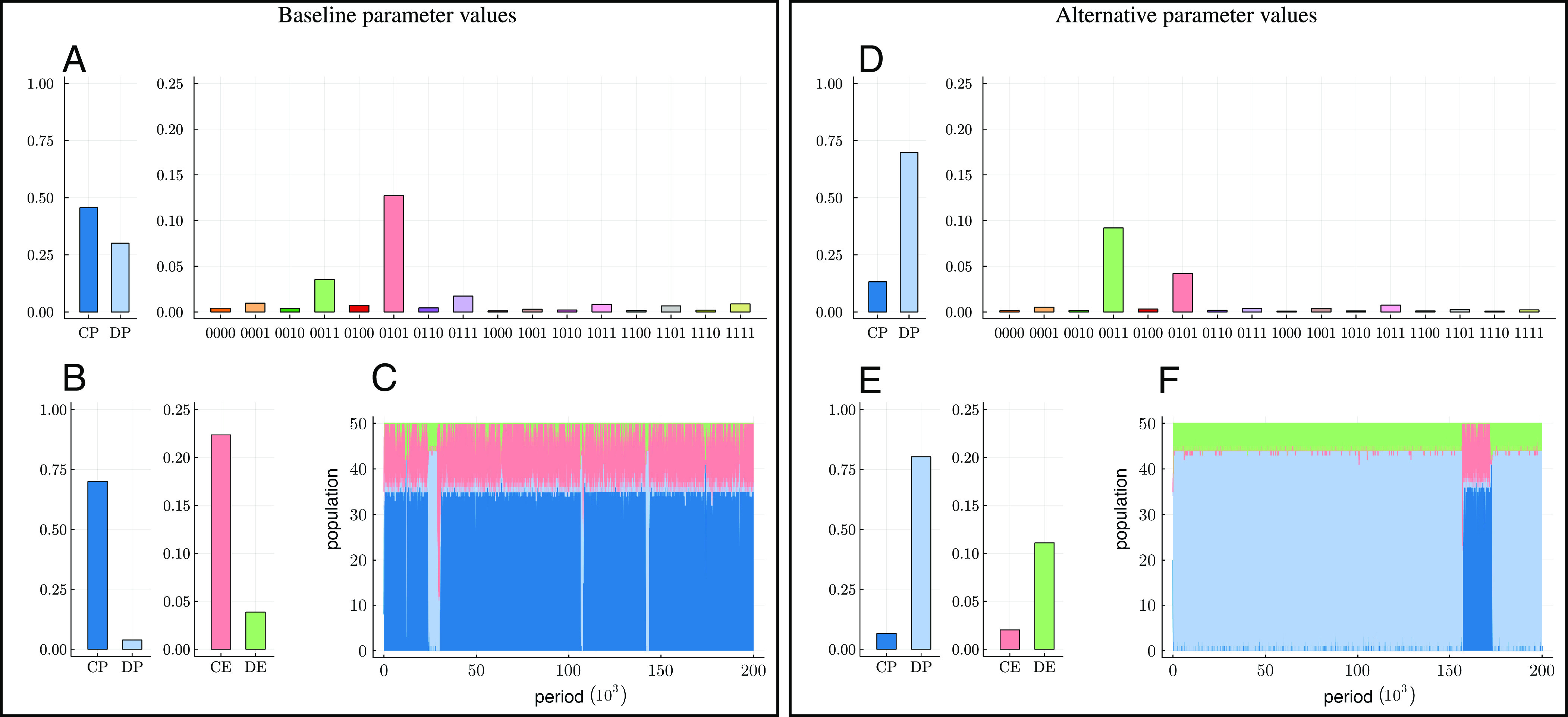
Shares of strategies in the invariant distribution and typical time series under baseline and alternative parameter values. Strategies are based on the CE standard. (*A* and *D*) Strategy shares when there are 16 enforcer strategies. (*B* and *E*) Strategy shares when there are 2 enforcer strategies. (*C* and *F*) Typical realization of the evolution of shares of strategies over 2 × 10^5^ iterations of the repeated game, with revision noise *ε* = 0.01. Invariant distributions are approximated by time averages over 10^6^ iterations for seven different, randomly drawn initial conditions. **Baseline** parameter values: *b* = 4, *c* = 1, *v* = 0.1, *f* = 0.3, *l* = 5. **Alternative** parameter values: *b* = 3, *c* = 2, *v* = 0.3, *f* = 0.4, *l* = 4. **Common** parameter values in both settings: *δ* = 0.9, *κ* = 8, *w* = 2, *τ* = 0.3, *p* = 2, *ψ* = 0.7, *μ* = 0.005, *η* = 0.01, *ε* = 0.05, (*γ*_*P*, *E*_, *γ*_*E*_, *γ*_*P*_)=(0.1, 0.3, 0.6).

We now turn to simulations to verify that the analytical conclusions hold under less-restrictive parameter assumptions and to explore further results. In order to approximate the invariant distribution, we simulate the learning process over many (10^6^) periods for different initial conditions and compute the time average of the shares of the different strategies. We define a baseline set of parameter values as our benchmark (Fig. 3 and *SI Appendix*, Table S1). We compare the baseline with an alternative set of parameter values. In the baseline setting, we have *b* = 4, *c* = 1, *f* = 0.3, *v* = 0.1, and *l* = 5, whereas in the alternative setting, *b* = 3, *c* = 2, *f* = 0.4, *v* = 0.3, and *l* = 4. All other parameters have the same values in the two specifications. Note that our baseline continuation probability, *δ* = 0.9, implies that the expected length of the repeated game is 10 rounds.

The analytical results presented above indicate that cooperation should be more difficult to attain in the alternative than in the baseline. We first consider the full set of 16 enforcer strategies, with the aim of selecting the most successful ones for further analysis. Fig. 3 *A* and *D* displays the results. In the baseline, CP dominates among the producers, whereas in the alternative setting, DP dominates. Among the enforcer strategies, the best performers are CE (coded 0101) and DE (0011) in both the baseline and the alternative.

Since mutations are drawn uniformly randomly from the entire set of strategies, the presence of a large number of enforcer strategies contributes to making the dynamic process more noisy. Reducing the mistake probability (“mutation rate”) would make the process less noisy but would also make it more difficult to approximate the invariant distribution. For this reason and in order to verify the robustness of our analytical results, we next remove all enforcer strategies except the top performers CE and DE. Fig. 3 *B* and *E* presents the results. In the baseline, CP dominates among producers and CE dominates among enforcers. In the alternative, DP dominates among producers and DE dominates among enforcers.

Our analytical results on stochastic stability relied on letting revision mistakes vanish. Then, the dynamic process will spend most of the time in, or near, the two equilibria. In Fig. 3 *C* and *F*, we display times series for the baseline and the alternative with a reduced amount of noise (*ε* = 0.01). It is clear that most of the time is spent in the equilibria, with occasional rapid shifts between them.

**Fig. 4.**
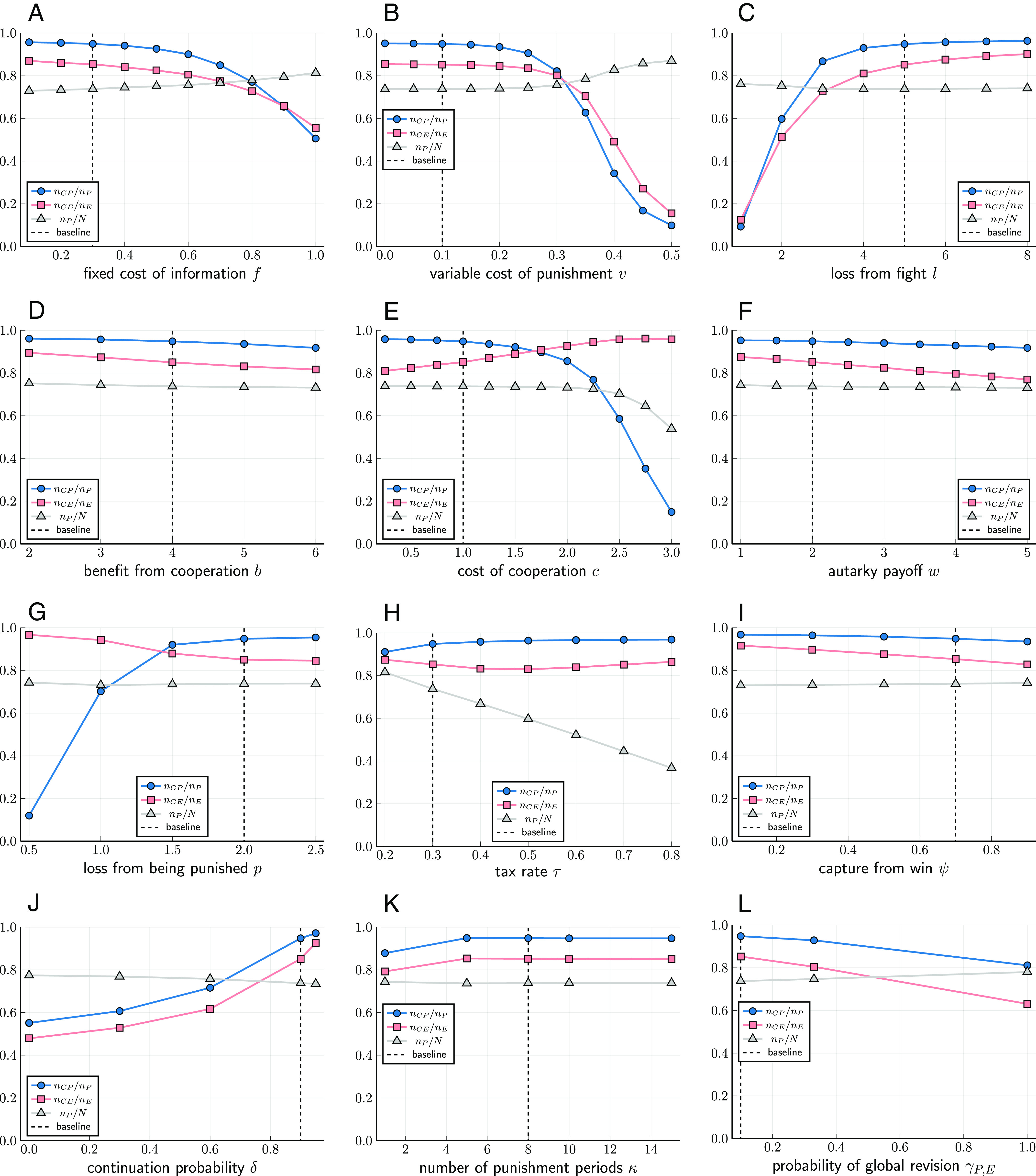
The time averages of *n*_*CP*_/*n*_*P*_ (circles), *n*_*CE*_/*n*_*E*_ (squares), and *n*_*P*_/*N* (triangles) for different values of parameters. Dotted vertical lines indicate baseline values. (*A*) fixed cost *f* of information; (*B*) variable cost *v* of punishment; (*C*) loss *l* from being attacked; (*D*) benefit *b* of cooperation; (*E*) cost *c* of cooperation; (*F*) autarky payoff *w*; (*G*) loss from being punished *p*; (*H*) tax rate *τ*; (*I*) gain to unilateral attacker *ψ*; (*J*) continuation probability *δ*; (*K*) length of punishment *κ*; (*L*) revision probabilities *γ*; we compare the baseline model, in which the vector of revision probabilities is (*γ*_*P*, *E*_,*γ*_*E*_,*γ*_*P*_) = (0.1,0.3,0,6), to the case of equally likely revision opportunities (1/3,1/3,1/3), and the case where all revision opportunities are maximally permissive (1,0,0).

We now turn to exploring the effect of each of the model’s parameters on the level of cooperation and the distribution of strategies. The stochastic stability analysis, Eq. **5**, indicates that the stability of cooperation is affected by the fixed cost of information *f* relative to the destructiveness of conflict between enforcers *l*. Recall that we set variable cost of punishment *v* = 0 in the analytical derivations. However, we expect *v* to have a similar effect as the fixed cost *f*. Fig. 4*A*–*F* confirms our predictions. Cooperation among producers is decreasing in *f*, *v*, and *c* and increasing in *l*. Changing *b* and *w* has limited effects on cooperation.

The analytical results state that, to deter defection among producers, the loss from punishment *p* needs to be large enough relative to *c*, which is in line with Fig. 4*G*. From Eqs. **3** and **4** we expect the tax rate *τ* to affect the equilibrium fractions of enforcers, which is confirmed in Fig. 4*H*.

For the cooperation equilibrium to exist, the continuation probability *δ* needs to be large enough (Fig. 4*J*). Only in this way may the temptation to attack an enforcer in good standing be outweighed by the future loss from ending up in bad standing. For the same reason, the length of the punishment phase *κ* needs to be large enough. However, a long punishment phase may have the drawback that CE agents who enter bad standing due to a mistake will remain in bad standing for a long time. Fig. 4*K* reveals that the effect of changing *κ* is limited (see also *SI Appendix*, section D in S4).

In the baseline model, the vector of revision probabilities is (*γ*_*P*, *E*_,*γ*_*E*_,*γ*_*P*_) = (0.1,0.3,0,6). We compare this to the case where all kinds of revision opportunities are equally likely, and the case where all revision opportunities allow both producer and enforcer strategies (Fig. 4*L*). The results are only mildly affected. In *SI Appendix*, Fig. S3, we report results indicating that the cooperation equilibrium is reasonably robust to small amounts of action mistakes (*μ*), logit imprecision (*η*), and revision mistakes (*ε*).

### Alternative Reputation System.

So far, we have assumed a single reputation system, with respect to which all strategies are defined. This is in line with much of the literature on indirect reciprocity; exceptions include (33, 36). Nevertheless, we wish to probe the robustness of our results when there is competition from other reputation systems. The CE reputation system used so far was defined in terms of the CE standard of behavior. As a counterpart, we now define a parochial enforcer (PE) reputation system, in terms of a PE standard of behavior. This standard does not care about actions against producers (unlike the CE standard), but it requires attacking a coplayer in the meta-enforcement step if and only if that coplayer is in bad standing (like the CE standard).

We define a PE strategy which never punishes in the enforcement step and punishes in the meta-enforcement step if and only if that coplayer is in bad standing. More generally, using this PE reputation system, we can define 2^4^ = 16 strategies, just as we did for the CE reputation system. For example, 0001 represents the PE strategy and 0011 is the DE strategy, which never punishes and always attacks, thereby ignoring the reputation system.

**Fig. 5.**
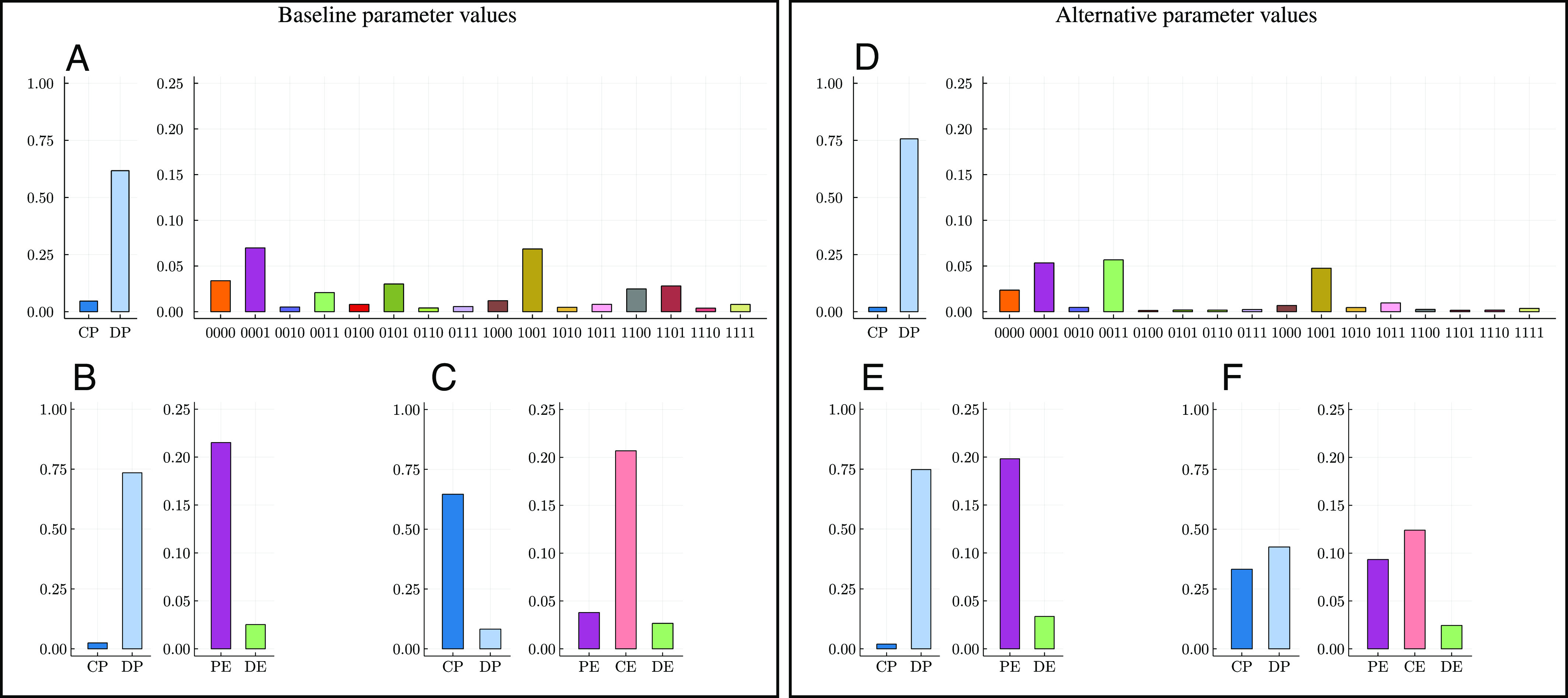
Shares of strategies in the invariant distribution under baseline and alternative parameter values. (*A* and *D*) Strategy shares when there are 16 enforcer strategies based on the PE standard. (*B* and *E*) Strategy shares when there are 2 enforcer strategies based on the PE standard. (*C* and *F*) Strategy shares when enforcer strategies are CE PE, and DE. Invariant distributions are approximated by time averages over 10^6^ iterations for seven different, randomly drawn initial conditions. **Baseline** parameter values: *b* = 4, *c* = 1, *v* = 0.1, *f* = 0.3, *l* = 5. **Alternative** parameter values: *b* = 3, *c* = 2, *v* = 0.3, *f* = 0.4, *l* = 4. **Common** parameter values in both settings: *δ* = 0.9, *κ* = 8, *w* = 2, *τ* = 0.3, *p* = 2, *ψ* = 0.7, *μ* = 0.005, *η* = 0.01, *ε* = 0.05, (*γ*_*P*, *E*_, *γ*_*E*_, *γ*_*P*_) = (0.1, 0.3, 0.6).

The results for the full set of sixteen enforcer strategies are presented in Fig. 5 *A* and *D*. Defection dominates among producers for both the baseline and the alternative parameter values. The enforcers are dominated by strategies PE, DE, and 1001. Next, we select the two best-performing enforcer strategies (strategy 1001 is excluded, since the only reason that it coexists with the PE strategy is that there are so few cooperators in the population). Fig. 5 *B* and *E* displays the results when DE and PE are the only enforcer strategies. Again, defection dominates among the producers, and PE dominates among the enforcers.

Finally, we add CE to the set of enforcer strategies. This means that there is competition between the two different reputation systems. The results are shown in Fig. 5 *C* and *F*. In the baseline, cooperation dominates among the producers, and CE dominates among the enforcers. In the alternative, there is slightly less cooperation than defection and more CE than other enforcer strategies, but the differences are smaller, as expected. In *SI Appendix*, Fig. S2, we repeat the analysis from Fig. 4 with PE added to the set of enforcer strategies. We conclude that the cooperative equilibrium supported by the CE strategy is robust to the inclusion of reputation systems that do not incentivize cooperation among producers.

## Discussion

Cooperation among producers is strongly affected by parameters governing the interactions between enforcers (*f*, *v*, and *l*), while only *c* has a clear effect among the parameters that govern producer–producer interactions (the effect of *b* is very small). Moreover, the severity of punishment *p* needs to be above a certain threshold for cooperation to take hold. By comparison, in models of direct and indirect reciprocity, cooperation is typically determined only by the costs and benefits of cooperation. In those models, cooperation among producers is incentivized by indefinite repetition of the producer interaction. By contrast, in our model of specialized reciprocity, cooperation among producers is incentivized by enforcers’ punishment of defectors which, in turn, is incentivized by indefinite repetition of the enforcer interaction.

From the perspective of the enforcers, punishing a DP can be viewed as contributing to a public good: It incentivizes cooperation among producers, which leads to a larger surplus that can be taxed by enforcers. For an individual enforcer, there is a temptation to avoid the cost of punishing, but this is detrimental to the future payoff of the enforcers. In the cooperation equilibrium, the enforcers manage to solve this second-order public goods provision problem by acting as a cohesive enforcement organization. The result is that in the cooperative equilibrium, both enforcers and producers earn higher payoffs than in the noncooperative equilibrium.

The extensive literature on the evolution of cooperation contains very few attempts at modeling third-party enforcement institutions, and, as far as we know, none as explicit as ours. Static models of third-party enforcement have been developed by, for example, refs. 10, 26, and 46, where enforcers are mainly disciplined by the fear of losing paying clients, and by ref. 47, where enforcers are disciplined by fear of contagion (21) rather than by reputation concerns.

Our aim has been to develop a stylized evolutionary-game-theoretic model that elucidates key mechanisms for how specialized third-party enforcement may emerge and be sustained. Consequently, the model is abstract and not tailored to any specific application. Still, it accurately describes important features of some real-world governance institutions, where i) there is specialization between producers and enforcers, ii) producers struggle to achieve cooperation in the absence of third-party involvement, and iii) enforcers discipline clients and other enforcers for violations of commonly held norms or conventions. In particular, we argue that the Sicilian mafia (25) and American prison gangs (48) function as enforcer organizations in our sense. They operate in environments where the state is unwilling or unable to enforce cooperation and where other decentralized forms of enforcement (based on direct or indirect reciprocity) are ineffective.

### Mafia.

While the mafia is typically associated with extortion and other criminal activities, Gambetta (25) (see also ref. 49) argues that the core activity of the Sicilian mafia is to provide private protection. Similar accounts have been given of organized crime in Russia (50) and Japan (51). Members of the mafia are specialized in information gathering and conduct of violence. They oversee (typically illegal) transactions and punish breaches of norms or implicit contracts. As an illustration, Gambetta (25, p.15) recounts what a cattle breeder in Palermo told him: “When the butcher comes to me to buy an animal, he knows that I want to cheat him [by giving him a low-quality animal]. But I know that he wants to cheat me [by reneging on payment]. Thus, we need Peppe [that is, a third party] to make us agree. And we both pay Peppe a percentage of the deal.” The mafia may exploit its clients (corresponding to a high tax rate in our model), but it does provide protection and enforcement services (as in the cooperative equilibrium).

Further, in line with the cooperative equilibrium of our model, which features peace among the enforcers, the mafiosi keep track of each other and sanction those who do not conform to the organization’s code of conduct. For instance, according to “the commandments” (25, p.147), stealing from other members of the mafia, and stealing in general, is forbidden. From the 1950s until the 1980s, the mafiosi’s (enforcers’) control of each other was formalized by the commissione, a loose association among Sicilian mafia families that “offered a forum for bargaining over market distribution, for seeking agreement on coordinated actions, and for upholding standards of behavior beneficial to the industry as a whole” (25, p.245). The foremost aim of the commissione in the latter context was to regulate the use of violence (among and within mafia families but also toward nonmembers) by requiring permission for killings and sanctioning those who violated these norms. Of course, at times, attempts to coordinate and cooperate among members of the mafia were not successful, resulting in periods of warfare (e.g., 1961–1963 and 1981–1985). This breakdown of cooperation is in line with our stochastic evolutionary model where the system may spend most of the time in an equilibrium but is occasionally interrupted by periods of instability, and there may be a transition to another equilibrium (as illustrated in Fig. 3 *C* and *F*).

### Prison Gangs.

Skarbeck (48) studies prison gangs in the Californian prison system, especially the so-called “Mexican mafia.” Prison gangs are ethnically segregated and, within their respective ethnic group, they function as the enforcers in our model. For instance, when a Hispanic member of a street gang (corresponding to a producer) arrives in prison, they seek the protection of the Mexican prison gang. Prison gangs also provide governance outside of prison by enforcing agreements and adjudicating disputes among street gangs. Much like the enforcers in our model, the prison gangs have an incentive to maintain order on the streets because they obtain revenue from taxation of drug dealers.

The Mexican prison gang has a constitution that governs relations among members. It specifies, among other things, that “a member must not raise a hand against another member without sanction” and “a member must not steal from another member” (48, p.118]. This corresponds closely to the CE standard of correct behavior in our model.

The gang-based governance order of the Californian (and more generally the US) prison system emerged after the 1960s, when the number of prisoners grew dramatically relative to the number of guards and the prisons became overcrowded. Previously, order was maintained both by the presence of prison guards and a private enforcement system based on personalized relationships and reputations (as in direct and indirect reciprocity). When the prison population grew, neither of these mechanisms could sustain the peace. Prison gangs emerged to provide enforcement services. Similar forces seem to be at play more generally in prisons across the globe (52).

### Further Applications.

On the other side of the law, bar associations in the Anglo-American legal system, with their self-regulatory functions, may be thought of as enforcers playing their part in the cooperative equilibrium of our model. Indeed, the New York City Bar Association, the oldest in the United States, was founded in 1870 by attorneys who were “anxious to do something about corruption at the bench and its consequences for the profession itself” (53, p. 357).

From a historical perspective, we speculate that the emergence of enforcement organizations from a situation without local monopolies of violence may have parallels in the formation of warrior elites in early Bronze Age Europe (54). During that era, being a warrior became a specialized occupation, partly due to the training needed to master new sets of weaponry. The period was also characterized by the emergence of stable long-distance alliances among elites. The increased lethality of military technology (corresponding to increased *l* in our model) may have been one of many factors that stimulated the development of such alliances.

## Materials and Methods

### Reputation System.

The CE and PE reputation systems are two different ways of extending Kandori’s reputation system (20) to our setting. They agree on how to condition treatment of enforcers on their standing, but they disagree on whether treatment of producers should matter for standing.

Each agent *i* has a score *z*_*i*_ ∈ {0,1,2,…,*κ*}, with the interpretation that if *z*_*i*_ = 0, then *i* is in good standing, and if *z*_*i*_ > 0, then *i* is in bad standing and shall be punished/attacked for the next following *z*_*i*_ rounds, including the current round. Scores are updated as follows: If an enforcer is in good standing (*z*_*i*_ = 0) and complies with the relevant (CE or PE) standard, she remains in good standing. If an enforcer does not comply with the standard, she enters bad standing with *z*_*i*_ = *κ*. If a player with *z*_*i*_ = *k* > 0 complies with the standard, her score is updated to *z*_*i*_ = *k* − 1. In case of action mistakes, scores are updated in accordance with actual actions taken.

### Subgame Perfect Nash Equilibrium Analysis.

Let s^*^ = (*s*^*CP*^,*s*^*CE*^) denote the strategy profile in which each producer *i* follows strategy CP, and each enforcer *i* follows strategy CE. We can then prove that if Eqs. **1** and **2** hold, there is some *κ* and some δ^*^ < 1 such that if *δ* ∈ (δ^*^,1), then s^*^ constitutes a subgame perfect Nash equilibrium (*SI Appendix*, Theorem S1). The theorem and proof are similar to Theorem 2 of ref. 20, adjusted for the fact that we have a sequential stage game, and two kinds of players: producers and enforcers.

### Payoffs.

Each agent *i*’s payoff in the repeated game is the average round-payoff she receives, i.e., $\sum_{t=1}^{T}\pi_{i}\left(t\right)/T$, where *π*_*i*_(*t*) is *i*’s payoff in round *t* and *T* is the realized number of rounds in that period. For our analytical results, we calculate payoffs under the limiting assumption of no action mistakes (*μ* = 0), infinite repetition (*δ* → 1), and infinite punishment (*κ* → ∞). We assume *τw* > *v*, which implies positive tax revenue *R*_*i*_ > 0. Under these assumptions, it is straightforward to derive expected payoffs of producers.

A DE ends up in bad standing as soon as she has been matched with a DP in the enforcement step or matched with a CE in the meta-enforcement step. She will then remain in bad standing for the rest of the period, since *κ* → ∞. Thus, the probability that all DE are in bad standing approaches one as the number of rounds that have been played grows (provided that there is at least one DP or one CE in the population). Moreover, as the number of rounds of the repeated game grows (as *δ* → 1), the fraction of rounds in which almost all DE are in bad standing goes to one (see *SI Appendix* for a formal argument). Hence, the average payoffs will be approximately equal to the payoff obtained when all CE are in good standing and all DE are in bad standing (section C in *SI Appendix*, S2).

### Exact Best-Reply Learning.

The evolutionary process employed for our dynamic analytical results is as follows. Starting from period 2, at the beginning of each of the following *n*_*P*_ periods, one producer is drawn at random (without replacement) and may choose among the producer strategies *S*^*P*^. Once all producers have decided on their strategy, one enforcer is drawn at random and may choose among the enforcer strategies *S*^*E*^. After this, the producers, one by one, again receive the opportunity to update their strategy, followed by an enforcer, and so on. Once all enforcers have updated their strategy, and all producers have had the opportunity to update following the last enforcer, one agent is selected at random from the whole population and may choose her strategy from the full set of strategies *S*^*P*^ ∪ *S*^*E*^. The process is repeated ad infinitum.

Under the best-response process, an agent assumes that the rest of the population do not revise their strategies. When a revision opportunity arises, the agent chooses the strategy that would have maximized her previous per-round expected payoff. The resulting unperturbed best-response dynamic has two absorbing sets of states. One set *E*^*C*^ corresponds to the cooperation equilibrium. The other set *E*^*D*^ corresponds to the defection equilibrium

$$\begin{array}{cc} E^{C} & =\{n\in N|n_{\mathrm{CP}}=n_{P},n_{\mathrm{CE}}=n_{E}\;\text{and}\;\alpha^{C}N-1\leq n_{\mathrm{CE}}\leq \alpha^{C}N\}\\ E^{D} & =\{n\in N|n_{\mathrm{DP}}=n_{P},n_{\mathrm{DE}}=n_{E}\;\text{and}\;\alpha^{D}N-1\leq n_{\mathrm{DE}}\leq \alpha^{D}N\}. \end{array}$$

Here, 𝒩 denotes the set of population states. Assuming *p* > (1−*τ*)*c* and *N* sufficiently large, the basins of attraction for the absorbing sets are characterized as follows (*SI Appendix*, Lemmas S1–S3).

1. From a state with $n_{\mathrm{CE}}>\frac{f}{l}\left(n_{E}-1\right)+1$, the process converges to *E*^*C*^ with probability one,
2. from a state with $n_{\mathrm{CE}}<\frac{f}{l}\left(n_{E}-1\right)$ or *n*_*E*_ < 2, the process converges to *E*^*D*^ with probability one, and
3. from a state with $\frac{n_{\mathrm{CE}}-1}{n_{E}-1}\leq \frac{f}{l}\leq \frac{n_{\mathrm{CE}}}{n_{E}-1}$ and *n*_*E*_ ≥ 2, the process converges to either *E*^*C*^ or *E*^*D*^ with positive probability.

### Stochastic Stability.

We analyze the invariant distribution in terms of stochastic stability (40–43). This technique effectively compares the number of revision mistakes (or “mutations”) that are required to move into and out of the different basins of attraction. As *ε* → 0, this distribution puts almost all weight on a single state. Such a state is called stochastically stable (41). We employ the notions of radius and coradius of ref. 43 to identify conditions under which each of the two equilibria is stochastically stable (*SI Appendix*, Theorem S2). Suppose *p* > (1−*τ*)*c*, and *f* < *l*. In a sufficiently large population, if Eq. **5** holds, then the cooperation equilibrium *E*^*C*^ is stochastically stable. If the inequality in Eq. **5** is reversed, then the defection equilibrium *E*^*D*^ is stochastically stable.

### Simulations and Figures.

In order to approximate the invariant distribution, we simulate the learning process for seven different (randomly drawn) initial conditions and compute the time average of the shares of the different strategies over the seven runs. We iterate the repeated game over 10^6^ periods (i.e., 10^6^ instances of the repeated game and equally many revisions), with one agent revising in each iteration, in a population of 50 agents. We verify that each of the seven different runs is close to the average (the largest SE is 0.024 and most are much smaller, with median value 4 × 10^−4^), indicating that we have found a good approximation of the invariant distribution. For a description of how we handle unmatched agents (in the case of an odd number of producers or an odd number of enforcers), see section A in *SI Appendix*, S4.

Noisy best responses are generated by a logit choice function, i.e., an individual picks strategy *X* from the strategy set *S* with probability

[6] $$\begin{array}{c} q_{S}\left(X\right)=\frac{\exp(\pi_{X}/\eta )}{\sum_{Y\in S}\exp\left(\pi_{Y}/\eta \right)}. \end{array}$$

The various *π*_*Y*_ are the realized average payoffs of *Y*-strategy agents in the current round.

## Supplementary Material

Appendix 01 (PDF)

## Data Availability

All code as well as the raw data used to create Figs. 3–5 and *SI Appendix*, Figs. S2 and S3 are publicly available on GitHub: https://github.com/alex-rigos/Community-Enforcement. All simulations and numerical calculations were performed with Julia 1.7.

## Footnotes

^*^To facilitate the exposition, we use the pronouns “she/her” for all agents throughout the article.
