# Supplementary material for: Emergence of specialized third-party enforcement
Source: Proc Natl Acad Sci U S A. 2023 Jun 6;120(24):e2207029120. doi: 10.1073/pnas.2207029120 (PMC10268577; doi:10.1073/pnas.2207029120)
Supplement: Supplementary file 1 — Appendix 01 (PDF) [file pnas.2207029120.sapp.pdf]

Supporting Information for

Emergence of Specialised Third-Party Enforcement

Erik Mohlin, Alexandros Rigos and Simon Weidenholzer

Erik Mohlin
E-mail: erik.mohlin@nek.lu.se

This PDF file includes:

Supporting text
Figs. S1 to S4
Table S1
SI References

Supporting Information Text

Methods

S1. Subgame Perfect Nash Equilibrium Analysis

A. The Repeated Game. Within each time period of the evolutionary model analysed in the main body of the paper, the members of the population are involved in an indefinitely repeated game. We now provide a standard non-evolutionary game-theoretic analysis showing that full cooperation is a subgame perfect Nash equilibrium of the repeated game. In the evolutionary model it is necessary to restrict attention to a relatively small set of strategies but in the following we impose no restrictions on the strategy sets, i.e. we analyse equilibria while allowing for all possible deviations. We consider the same repeated game as in the main text. Recall that it consists in the indefinite repetition of the stage game consisting of the following three steps:

1. *Production*: Each producer pair plays a Prisoner's Dilemma (PD).

2. *Enforcement*: Each enforcer may punish and/or take resources (tax) from her clients.

3. *Meta-Enforcement*: Each enforcer may try to take resources from her matching partner.

Before playing the repeated game the enforcer players can choose whether to pay the cost f to be informed about their clients' actions and the other enforcers' reputations. Players who do not pay this cost are unable to use repeated game strategies that condition on producer behaviour or enforcer reputation. In what follows, $\mathcal{N}_P$ and $\mathcal{N}_E$ denote the sets of producers and enforcers, respectively.

Actions The action set of a producer in step 1 (production) is $\mathcal{A}_1 = \{C, D\}$. The action set of an enforcer facing a producer in step 2 (enforcement) is $\mathcal{A}_2 = \{P, Q\}$, where P denotes punishment, and Q denotes no punishment. The action set of an enforcer facing another enforcer in step 3 (meta-enforcement) is $\mathcal{A}_3 = \{A, B\}$.

Histories The history of a producer in a stage game consists of the action profile in the producer's match in step 1 and the action by the enforcer in the producer's match in step 2, as well as the identities of the enforcer and the other producer. The set of all such stage game histories is $\mathcal{H}_P = \mathcal{A}_1 \times \mathcal{A}_1 \times \mathcal{A}_2 \times \mathcal{N}_P \times \mathcal{N}_E$. The set of possible histories of a producer at the beginning of step 1 of the stage game in round r is $\mathcal{H}_P(r) = \mathcal{H}_P^{r-1}$. The set of all possible histories of a producer at the beginning of step 1 of some stage game is $\mathcal{H}_P = \cup_{r=1}^{\infty} \mathcal{H}_P(r)$.

Let M_1 be a symmetric $n^P \times n^P$ matrix such that entry ij is equal to 1 if producers i and j are matched and 0 otherwise, and let $\mathcal{M}_1$ denote the set of all such matrices, i.e all possible matchings in step 1 of the stage game. Let M_2 be an $n^P \times n^E$ matrix such that entry ij is equal to 1 if i is a client of j and 0 otherwise, and let $\mathcal{M}_2$ denote the set of all such matrices, i.e. all possible matchings in step 2 of the stage game. Let M_3 be a symmetric $n^E \times n^E$ matrix such that entry ij is equal to 1 if enforcers i and j are matched and 0 otherwise, and let $\mathcal{M}_3$ denote the set of all such matrices, i.e. all possible matchings in step 3 of the stage game.

The history of an enforcer in a stage game consists of all actions taken in the game, as well as the matching of all steps. The set of all such stage game histories is $\mathcal{H}_E = \mathcal{A}_1^{n_P} \times \mathcal{A}_2^{n_E} \times \mathcal{A}_3^{n_E} \times \mathcal{M}_1 \times \mathcal{M}_2 \times \mathcal{M}_3$. The set of possible histories of an enforcer at the beginning of step 2 of the stage game in round r is $\mathcal{H}_{E2}(r) = \mathcal{A}_1^{n_P} \times \mathcal{M}_1 \times \mathcal{H}_E^{r-1}$. The set of all possible histories of an enforcer when in step 2 of some stage game is $\mathcal{H}_{E2} = \cup_{r=1}^{\infty} \mathcal{H}_{E2}(r)$. The set of possible histories of an enforcer at the beginning of step 3 of the stage game in round r is $\mathcal{H}_{E3}(r) = \mathcal{A}_1^{n_P} \times \mathcal{A}_2^{n_E} \times \mathcal{M}_1 \times \mathcal{M}_2 \times \mathcal{H}_E^{r-1}$. The set of all possible histories of an enforcer when in step 3 of some stage game is $\mathcal{H}_{E3} = \cup_{r=1}^{\infty} \mathcal{H}_{E3}(r)$.

Strategies A pure strategy of producer i, denoted s_i^P specifies whether to play C or D in the current round, as a function of history; $s_i^p : \mathcal{H}_P \to \{C, D\}$. The set of all such strategies is S^P.

A pure strategy of enforcer i, is a tuple $s_i^E = \left(s_i^{E2}, s_i^{E3}\right)$, where $s_i^{E2} : \mathcal{H}_{E2} \times \mathcal{N}_P \to \{P, Q\}$ specifies whether the enforcer should punish a client in the enforcement step, as a function of history and the identity of the client, and $s_i^{E3} : \mathcal{H}_{E3} \to \{A, B\}$ specifies whether the enforcer attacks or not in the meta-enforcement step, as a function of history.

B. Cooperative Equilibrium.

Reputation We will define our cooperative strategy profile with the help of a reputation system, as follows. Each agent i has a label $z_i \in \{0, 1, 2, \ldots, \kappa\}$, with the interpretation that if $z_i = 0$ then i is in good standing and if $z_i = k > 0$ then i is in bad standing and shall be punished/attacked for the next following k rounds, including the current round. Labels are updated as follows: If an agent is in good standing ($z_i = 0$) and follows the proposed strategy, then she remains in good standing. If an agent does not follow the proposed strategy then she becomes enters bad standing with $z_i = \kappa$. If a player with $z_i = k > 0$ follows the proposed strategy then her label is updated to $z_i = k - 1$. Thus, if a player with $z_i = 1$ follows the proposed strategy then she becomes enters good standing ($z_i = 0$).

Note that since enforcers know the complete history (know all actions taken by everyone in the repeated game so far) they can derive the label of any enforcers.

Equilibrium Let $s^* = \left(s^{CP}, s^{CE}\right)$ denote the strategy profile in which each producer i follows strategy CP, and each enforcer i follows strategy CE. We can show that s^* is a subgame perfect Nash equilibrium if the continuation probability δ is high enough, and the punishment phase for enforcers is long (κ large) enough, provided that the punishment p of producers is severe enough. This result and the proof of it (except the part concerning unconditional strategies) is similar to Theorem 2 of (1), adjusted for the fact that we have a sequential stage game, and two kinds of players, producers and enforcers.

We perturbed the environment by assuming that producers make a mistake and fail to play their intended action with probability μ_P (i.i.d. across players and rounds) and look for subgame perfect Nash equilibria as $\mu_P \to 0$.

Theorem S1 *Suppose that $l > 2\max\{f, v\}$ and $(1-\tau)\,c < p$. There are μ_P^*, κ^*, and δ^* such that if $\mu_P < \mu_P^*$, $\kappa = \kappa^*$, and $\delta \in (\delta^*, 1)$, then s^* constitutes a subgame perfect Nash equilibrium.*

Proof. First consider **producers**. Ignore mistakes for now. A producer complying with s^{CP} earns $(1-\tau)(b-c+w)$ in a round in which she faces a cooperating co-player and $(1-\tau)(-c+w)$ in a round in which she faces a defecting co-player. If she would deviate from s^{CP} and play D instead of C she would earn $(1-\tau)(b+w)-p$ in a round in which she faces a cooperating co-player and $(1-\tau)(w)-p$ in a round in which she faces a defecting co-player. Her deviation does not affect the behaviour of other players. Thus deviation to defection is unprofitable if $(1-\tau)\,c < p$. This is true for a any subgame since the strategy s^{CE} always prescribes punishment of defection. The above argument continues to hold in the presence of sufficiently small mistakes, since all the inequalities governing profitability are strict.

Next we examine whether **enforcer** i has any profitable deviation from the proposed strategy, given that the remaining players comply with the strategy. For ease of exposition we assume that $n_E = 2n_P$. This is without loss of generality.

Recall that strategies that do not incur the fixed cost f are unable to condition on whether a producer defected or not and unable to condition on whether an enforcer is in good or bad standing. To begin with we exclude these strategies and show that it is not profitable to deviate to a strategy that has paid the fixed cost f and conditions on producer behaviour or enforcer standing. In this case we can utilise the *one-shot deviation principle*, which states that a strategy profile of a repeated game (with $\delta < 1$) is a subgame perfect Nash equilibrium if and only if the following holds: At every information set, the player acting there cannot increase her payoff by deviating at that information set and then returning to her strategy for the rest of the game, given that all other players stick to their strategies throughout the game. The one-shot deviation principle is not valid when we examine deviations to strategies that cannot condition on the same events, so we perform that comparison separately.

Strategies paying the fixed cost f: We ignore mistakes, noting that the argument presented below continues to hold in the presence of sufficiently small mistakes, since all the inequalities governing profitability are strict.

Note that regardless of whether other players are in good or bad standing they will behave in the same way towards i, since they are assumed to comply with the proposed strategy, and the proposed strategy only conditions behaviour on the label of the co-player and not on one's own label. Also note that if all players start complying with the proposed strategy profile in round 1, then from round $\kappa+1$ and onwards everyone is in good standing. In what follows let the action that the proposed strategy profile prescribes for player i in the meta-enforcement step of round t be denoted a_i^t, i.e.

$$a_i^t = \begin{cases} B & \text{if } i \text{ faces a player in good standing in round } t, \\ A & \text{if } i \text{ faces a player in bad standing in round } t. \end{cases}$$

Let $R^{t,comp}$ be the the maximum amount of resources obtained in the enforcer step by an enforcer who punish defections in round t, and let $R^{t,dev}$ be the maximum amount of resources that can be obtained in the enforcer step by an enforcer who does not punish defections in round t. Note that

$$R^{t,dev} \leq \tau \max\{2(b-c+w), 2w, b-c+2w\} = \tau 2(b-c+w),$$

and

$$R^{t,dev} - R^{t,comp} \leq 2v.$$

We define the dummy variables A_i^t and B_i^t as follows (note that $B_i^t = 1 - A_i^t$):

$$A_i^t = \begin{cases} 1 & \text{if } a_i^t = A \\ 0 & \text{if } a_i^t = B \end{cases} \qquad B_i^t = \begin{cases} 0 & \text{if } a_i^t = A \\ 1 & \text{if } a_i^t = B \end{cases}$$

There are $\kappa+1$ different kinds of subgames to consider, corresponding to $z_i \in \{0, 1, 2, \ldots, \kappa\}$.

Case κ: If i is in bad standing with $z_i = \kappa$ her payoff from complying with the proposed strategy in both the enforcement and meta-enforcement steps is at least

$$(1-\psi)\,R^{1,comp} - l + \sum_{t=2}^{\kappa} \delta^{t-1}\left(\left(1 - B_i^t\psi\right)2\tau(b-c+w) - l\right) + \sum_{t=\kappa+1}^{\infty} \delta^{t-1} 2\tau(b-c+w), \qquad \text{[S1]}$$

since from round $\kappa+1$ and onwards everyone is in good standing. Her payoff from deviating (one-shot deviation in round one) is at most

$$R^{1,dev} - l + \sum_{t=2}^{\kappa} \delta^{t-1}\left(\left(1 - B_i^t\psi\right)2\tau(b-c+w) - l\right) + \delta^{\kappa}\left((1-\psi)\,2\tau(b-c+w) - l\right) + \sum_{t=\kappa+2}^{\infty} \delta^{t-1} 2\tau(b-c+w). \qquad \text{[S2]}$$

Again we use the fact that from round $\kappa+1$ and onwards everyone is in good standing, but i remains in bad standing until the end of round $\kappa+1$. Note that

$$\begin{aligned}(1-\psi)\,R^{1,comp}-R^{1,dev} &> (1-\psi)\,R^{1,comp}-\left(R^{1,comp}+2v\right)\\ &= -\psi R^{1,comp}-2v\\ &> -2v-\psi\tau 2\,(b-c+w)\,.\end{aligned}$$

Using this observation when subtracting (S2) from (S1) we find that deviation is unprofitable if

$$-2v-\psi\tau 2\,(b-c+w)+\delta^{\kappa}\left(\psi 2\tau\,(b-c+w)+l\right)>0. \tag{S3}$$

Note that as $\delta^{\kappa}\to 1$ the left hand side of (S3) approaches

$$-2v-\psi\tau 2\,(b-c+w)+\psi 2\tau\,(b-c+w)+l=-2v+l.$$

Thus if $l>2v$ then (S3) is satisfied as $\delta^{\kappa}\to 1$.

Case $1,2,3,...,\kappa-1$: If i is in bad standing with $z_i<\kappa$ her payoff from complying with the proposed strategy in both the enforcement and meta-enforcement steps is at most,

$$\begin{aligned}&(1-\psi)\,R^{1,comp}-l+\sum_{t=2}^{z_i}\delta^{t-1}\left(\left(1-B_i^t\psi\right)2\tau\,(b-c+w)-l\right)\\ &+\sum_{t=z_i+1}^{\kappa}\delta^{t-1}\left(1+\psi A_i^t\right)2\tau\,(b-c+w)+\sum_{t=\kappa+1}^{\infty}\delta^{t-1}2\tau\,(b-c+w)\,,\end{aligned}$$

which is strictly higher than the payoff of complying in the case of $z_i=1$. In contrast her payoff from deviating (one-shot deviation in round one) is the same as in the case of $z_i=1$. Thus, if (S3) holds then a agent i with $z_i>1$ will also find it unprofitable to deviate.

Case 0: If i is in good standing, $z_i=0$, her payoff from complying with the proposed strategy is

$$R^{1,comp}+\sum_{t=2}^{\kappa+1}\delta^{t-1}\left(1+\psi A_i^t\right)2\tau\,(b-c+w)+\sum_{t=\kappa+2}^{\infty}\delta^{t-1}2\tau\,(b-c+w)\,. \tag{S4}$$

Deviation may begin in the enforcement step and in the in the meta-enforcement step. Let $R^{1,\max}$ denote the highest payoff that any of these deviations can yield, so that the payoff from deviating (one-shot deviation), is at most

$$R^{1,\max}+\sum_{t=2}^{\kappa+1}\delta^{t-1}\left(\left(1-B_i^t\psi\right)2\tau\,(b-c+w)-l\right)+\sum_{t=\kappa+2}^{\infty}\delta^{t-1}2\tau\,(b-c+w)\,. \tag{S5}$$

Note that

$$\left(1+\psi A_i^t\right)2\tau\,(b-c+w)-\left(\left(1-B_i^t\psi\right)2\tau\,(b-c+w)-l\right)\geq l.$$

Thus, subtracting (S5) from (S4) we find that deviation is unprofitable if

$$R^{1,comp}-R^{1,\max}+\left(\delta+\delta^2+\ldots\delta^{\kappa}\right)l\geq 0.$$

Multiplied by $1-\delta$ this becomes

$$(1-\delta)\left(R^{1,comp}-R^{1,\max}\right)+l\delta\,(1-\delta^{\kappa})\geq 0. \tag{S6}$$

Note that for a given value of δ^{κ}, if $\delta\to 1$ then the left hand side goes to

$$l\,(1-\delta^{\kappa})>0.$$

Cases 0 and 1 together: We now show that (S3) and (S6) can be satisfied for some δ and some κ. We have noted that condition (S3) holds for δ^{κ} sufficiently close to one. Fixing δ^{κ} and letting $\delta\to 1$ the left hand side of (S6) becomes strictly positive as we have noted. More precisely, there is some $\nu\in(0,1)$ such that if $\delta^{\kappa}\geq\nu\iff\kappa\leq\log\nu/\log\delta$ then (S3) holds. Set $\kappa=\log\nu/2\log\delta$ implying that $\delta^{\kappa}>\nu$ for any choice of δ, thereby satisfying (S3), and let $\delta\to 1$ so that (S6) is satisfied.

Strategies not paying the fixed cost f:

Fix $\mu_P>0$. The probability that at least one of two clients defect in a given round is at least $1-(1-\mu_P)^2$. Divide time into blocks with a lenght of $\kappa/2$ rounds. Fix an enforcer. Let K_t be random variable that takes the value 1 if the enforcer in question faces at least one defecting client in the tth block of $\kappa/2$ rounds, and takes the value 0 otherwise. The probability that an enforcer faces at least one defecting client in a block of $\kappa/2$ rounds is at least $\left((1-\mu_P)^2\right)^{\kappa/2}=(1-\mu_P)^{\kappa}$ It follows that for any $\varepsilon_A\in(0,1)$ there is some $\kappa^*_{\varepsilon_A}$ such that if $\kappa>\kappa^*_{\varepsilon_A}$ then the probability that an enforcer faces at least one defecting client in a block of $\kappa/2$ rounds is at least $1-\varepsilon_A$, i.e.

$$\kappa>\kappa^*_{\varepsilon_A}\Rightarrow\Pr\left(K_t=1\right)>1-\varepsilon_A.$$

Note that $\{K_t\}_{t=1}^{\infty}$ is a sequence of i.i.d. random variables. Let k be the number of blocks. By the weak law of large numbers the average $\bar{K} = \sum_{t=1}^{k} K_t/k$ converges in probability to $\mathbb{E}[K_t] = \Pr(K_t = 1)$ as $k \to \infty$. That is, for any $\varepsilon_B \in (0,1)$ and $\varepsilon_C \in (0,1)$ there is some $k^*_{\varepsilon_B,\varepsilon_C}$ such that

$$k > k^*_{\varepsilon_B,\varepsilon_C} \Rightarrow \Pr\left(\left|\bar{K} - \Pr(K_t = 1)\right| < \varepsilon_B\right) > 1 - \varepsilon_C,$$

and consequently

$$\kappa > \kappa^*_{\varepsilon_A} \wedge k > k^*_{\varepsilon_B,\varepsilon_C} \Rightarrow \Pr\left(\bar{K} > 1 - \varepsilon_A - \varepsilon_B\right) > 1 - \varepsilon_C.$$

Let T denote the number of rounds in the repeated game. The number of blocks of $\kappa/2$ rounds is $\lfloor 2T/\kappa \rfloor$. Let $\mathbb{M}_\delta(T)$ denote the median number of rounds given constant repetition probability δ. Note that T has a geometric distribution so that there is some $\delta^*_{\varepsilon_A,\varepsilon_B,\varepsilon_C}$ such that if $\delta > \delta^*_{\varepsilon_A,\varepsilon_B,\varepsilon_C}$ then $\left\lfloor 2\mathbb{M}_\delta(T)/\kappa^*_{\varepsilon_A} \right\rfloor > k^*_{\varepsilon_B,\varepsilon_C}$. By the definition of the median $\Pr(T > \mathbb{M}_\delta(T)) \geq 1/2$. It follows that

$$\delta > \delta^*_{\varepsilon_A,\varepsilon_B,\varepsilon_C} \Rightarrow \Pr\left(k > k^*_{\varepsilon_B,\varepsilon_C}\right) \geq 1/2,$$

and furthermore

$$\delta > \delta^*_{\varepsilon_A,\varepsilon_B,\varepsilon_C} \Rightarrow \Pr\left(\bar{K} > 1 - \varepsilon_A - \varepsilon_B\right) > (1 - \varepsilon_C)/2.$$

Consider an enforcer who has not invested in information networks (saving on the fixed cost f) and never punishes defecting clients. If $\kappa > \kappa^*(\varepsilon_A)$ and $\delta > \delta^*$ then with probability of at least $(1-\varepsilon_C)/2$ the enforcer will enter bad standing or restart bad standing in a at least a fraction $1 - \varepsilon_A - \varepsilon_B$ of the blocks of $\kappa/2$ rounds. Thus, with probability of at least $(1-\varepsilon_C)/2$ the enforcer will spend at least a fraction $1 - \varepsilon_A - \varepsilon_B$ of all rounds in bad standing.

The payoff to i of complying in a subgame at least as high as the payoff from complying in in a subgame where i is in bad standing with $z_i = \kappa$, i.e. at least

$$\pi^{com} = (1-\psi) R^{1,comp} - l + \sum_{t=2}^{\kappa} \delta^{t-1} \left((1-\psi) 2\tau (b - c + w) + o(\mu_P) - l\right)$$
$$+ \sum_{t=\kappa+1}^{\infty} \delta^{t-1} \left(2\tau (b - c + w) + Q\right).$$

where

$$Q := \left(1 - (1-\mu_P)^2\right) 2 \left(\tau (c - b) - v\right).$$

If $\kappa > \kappa^*$ and $\delta > \delta^*$ then the payoff from a strategy that never punishes defection is at most

$$\pi^{dev} = R^{1,dev} - l + \sum_{t=1}^{\kappa+1} \delta^{t-1} \left(2\tau (b - c + w) - l\right)$$
$$+ \sum_{t=\kappa+1}^{\infty} \delta^{t-1} \left[2\tau (b - c + w) - l \left(\frac{1-\varepsilon_C}{2}\right)(1 - \varepsilon_A - \varepsilon_B)\right].$$

Note that

$$\begin{aligned}
(1-\delta)\pi^{com} - (1-\delta)\pi^{dev} &\to (1-\delta)\delta^{t-1} \sum_{t=\kappa+1}^{\infty} \delta^{t-1} \left[2\tau (b - c + w) + Q\right] \\
&\quad - (1-\delta) \sum_{t=\kappa+1}^{\infty} \delta^{t-1} \left[2\tau (b - c + w) - l \left(\frac{1-\varepsilon_C}{2}\right)(1 - \varepsilon_A - \varepsilon_B)\right] \\
&= (1-\delta)\delta^{\kappa} \sum_{t=1}^{\infty} \delta^{t-1} (Q + l) \left(\frac{1-\varepsilon_C}{2}\right)(1 - \varepsilon_A - \varepsilon_B) \\
&= \delta^{\kappa} \frac{1}{2} (Q + l)(1 - \varepsilon_C)(1 - \varepsilon_A - \varepsilon_B).
\end{aligned}$$

Since $\frac{l}{2} > f$ we can pick μ_P such that $\frac{1}{2}(Q + l) > f$ and then pick $\varepsilon_A, \varepsilon_B, \varepsilon_C$ such that $\frac{1}{2}(Q + l)(1 - \varepsilon_C)(1 - \varepsilon_A - \varepsilon_B) > f$. Then pick $\kappa > \kappa^*_{\varepsilon_A}$. Finally pick $\delta > \delta^*_{\varepsilon_A,\varepsilon_B,\varepsilon_C}$ such that

$$\delta^{\kappa} \frac{1}{2} (Q + l)(1 - \varepsilon_C)(1 - \varepsilon_A - \varepsilon_B) > f.$$

This establishes that at every subgame a strategy that does not pay f and hence always fails to punish a defection with some arbitrarily small probability μ_P earns less than strategies that do pay f, provided that the punishment phase is long enough, and that the repetition probability is high enough (relative to the punishment phase). ■

S2. Analytical Results for Dynamics

A. Assumptions. In order to obtain analytical results, we necessarily need to make a number of simplifying assumptions. We only consider the CE and DE strategies for the enforcers, in addition to the CP and DP strategies for the producers. If the population is of size N then the set of states is

$$\mathcal{N} = \left\{ n \in \mathbb{N}^4 : n_{CP} + n_{DP} + n_{CE} + n_{DE} = N \right\}.$$

We abstract away from action mistakes and variable cost (by setting $\mu = v = 0$) and focus on the limit where the (expected) number of rounds per period and the number of punishment rounds get arbitrarily large ($\delta \to 1$ and $\kappa \to \infty$). Furthermore, we assume that punishment is sufficiently severe, $p > (1-\tau)\,c$, and sufficiently cheap, $f < l$. We assume $\tau w > v$, which implies positive tax revenue $R_i > 0$.

We define our strategies such that if $n_P = 0$ then the DE still attacks her co-player in the enforcement step, and the CE still has to pay the fixed cost. Reverting these assumptions do not change our results.

B. Remarks on Our Dynamics. The speed of convergence to equilibria can be increased dramatically by making interactions local (2, 3). Local interactions would also be perfectly realistic. However, we know that imposing spatial structure and local interactions is favourable for the evolution of cooperation, and hence would confound our results, making it unclear whether cooperation emerged due to specialised enforcement or due to local interactions.

We have examined the relative stability of the cooperation and defection equilibria by means of stochastic stability analysis. Alternatively one may consider the effect of group selection on which equilibrium is likely to be observed. It is plausible to assume that groups that manage to coordinate on the more efficient cooperation equilibrium will be favoured relative to groups that coordinate on the less efficient defection equilibrium, as suggested by (4) for the case of so-called altruistic peer-punishment. However, the introduction of several groups also opens up the possibility for new enforcer strategies, including those that try to tax or plunder the producers of other populations. For this reason an examination of the effect of group selection will have to be relegated to future research.

In our model we assume that revision across professions (i.e. across producer and enforcer roles) occur more rarely than revisions within professions. One could object that enforcers will have an incentive to limit entry into the enforcement business, thereby maintaining higher payoff for themselves. Such an attempt by enforcers can be modelled by adding a fixed cost of becoming an enforcer. It will reduce the fraction of enforcers in equilibrium but will not substantially alter the conclusions.

C. Payoff Calculations. We calculate payoffs under the limiting assumption of no action mistakes ($\mu = 0$), no monitoring mistakes ($\rho = 0$), infinite repetition ($\delta \to 1$), and infinite punishment ($\kappa \to \infty$). For now we allow for $v > 0$, though will (for reasons of tractability) set $v = 0$ in the stochastic stability analysis below.

C.1. Production. Let us first consider states where there are at least two producers, and at least one enforcer present in the population ($n_P \geq 2$ and $n_E \geq 1$). Consider a cooperative producer. When she faces a cooperating co-player her payoff is $(1-\tau)(b-c+w)$ and when faces a defecting co-player her payoff is $(1-\tau)(w-c)$, independently of whether she is matched with a DE or CE. In total the expected payoff of a cooperating producer is

$$\begin{aligned} \pi_{CP} = &\underbrace{\frac{n_{DP}}{n_P - 1}}_{\Pr(\text{Opponent defects})} \times (1-\tau)(w-c) \\ + &\underbrace{\frac{n_{CP}-1}{n_P - 1}}_{\Pr(\text{Opponent cooperates})} \times (1-\tau)(b-c+w) \\ = &(1-\tau)\left(w - c + \frac{n_{CP}-1}{n_P-1} b\right). \end{aligned}$$

Next consider a defecting producer. Her payoff in the production step is 0 when she faces another defector, and b when she faces a cooperating co-player. Regardless of the kind of enforcer she is matched with she has background resources w and is taxed at rate τ. If she faces a DE she is not punished but if she faces a CE she is punished, thereby suffering a loss of p. In total the expected payoff of a defecting producer is

$$\begin{aligned} \pi_{DP} = &\underbrace{\frac{n_{DP}-1}{n_P - 1}}_{\Pr(\text{Opponent defects})} \times \left(\underbrace{\frac{n_{DE}}{n_E}}_{\Pr(\text{Enforcer is DE})} (1-\tau)\,w + \underbrace{\frac{n_{CE}}{n_E}}_{\Pr(\text{Enforcer is CE})} ((1-\tau)\,w - p) \right) \\ + &\underbrace{\frac{n_{CP}}{n_P - 1}}_{\Pr(\text{Opponent cooperates})} \times \left(\underbrace{\frac{n_{DE}}{n_E}}_{\Pr(\text{Enforcer is DE})} (1-\tau)(b+w) + \underbrace{\frac{n_{CE}}{n_E}}_{\Pr(\text{Enforcer is CE})} ((1-\tau)(b+w) - p) \right) \\ = &(1-\tau)\left(w + \frac{n_{CP}}{n_P-1} b\right) - \frac{n_{CE}}{n_E} p. \end{aligned}$$

Now let us consider states where there is a single producer, i.e. $n_P = 1$ (implying $n_E \geq 1$ for $N > 2$) then there is no pair of producers that can play the PD so

$$\pi_{CP} = \pi_{DP} = (1 - \tau)\, w.$$

Finally, if there are no enforcers, $n_E = 0$, then we have

$$\pi_{CP} = w - c + \frac{n_{CP} - 1}{n_P - 1} b$$

and

$$\pi_{DP} = w + \frac{n_{CP}}{n_P - 1} b.$$

C.2. Enforcement. First, assume that at least one enforcer and at least two producers are present in the population ($n_E \geq 1$ and $n_P \geq 2$) so that there is at least one producer-producer interaction. Consider a cooperation enforcer and suppose she is matched with only one pair of producers. The expected payoff the enforcer obtains from one pair is

$$\begin{aligned}\pi_{CE|1} = & \underbrace{\frac{n_{DP}}{n_P}}_{\Pr(\text{Client defects})} \underbrace{\frac{n_{DP}}{n_P}}_{\Pr(\text{Client defects})} \times 2\,(\tau w - v) \\ & + 2 \underbrace{\frac{n_{CP}}{n_P}}_{\Pr(\text{Client cooperates})} \underbrace{\frac{n_{DP}}{n_P}}_{\Pr(\text{Client defects})} \times (\tau\,(w - c) + \tau\,(b + w) - v) \\ & + \underbrace{\frac{n_{CP}}{n_P}}_{\Pr(\text{Client cooperates})} \underbrace{\frac{n_{CP}}{n_P}}_{\Pr(\text{Client cooperates})} \times 2\tau\,(w + b - c) \\ & = 2\tau \left(w + \frac{n_{CP}}{n_P}\,(b - c)\right) - 2v \frac{n_{DP}}{n_P}.\end{aligned}$$

The expected number of client pairs per enforcer is $\frac{1}{2}\frac{n_P}{n_E}$, so the expected payoff from the enforcement stage is

$$\begin{aligned}\pi_{CE(E)} &= \frac{1}{2}\frac{n_P}{n_E}\pi_{CE|1} \\ &= \frac{n_P}{n_E}\tau \left(w + \frac{n_{CP}}{n_P}(b - c)\right) - v\frac{n_{DP}}{n_E} - f.\end{aligned}$$

Consider a defection enforcer. She receives the same payoff as a cooperation enforcer, but does not have to incur the cost v;

$$\pi_{DE(E)} = \frac{n_P}{n_E}\tau \left(w + \frac{n_{CP}}{n_P}(b - c)\right).$$

Now let us consider states where $n_E < 1$ or $n_P < 2$. If $n_P = 1$ (implying $n_E \geq 1$) then there is no pair of producers that can play the PD, so

$$\begin{aligned}\pi_{DE(E)} &= \tau w, \\ \pi_{CE(E)} &= \tau w - f.\end{aligned}$$

If $n_P = 0$, then

$$\begin{aligned}\pi_{DE(E)} &= 0, \\ \pi_{CE(E)} &= -f.\end{aligned}$$

C.3. Reputation. We have argued heuristically that as $\delta \to 1$ the fraction of rounds in which almost all DE are in bad standing goes to one. The argument can be formalised as follows. Let β be the fraction of DE who are in bad standing. A DE ends up in bad standing as soon as she has been matched with a DP in the production step or matched with a CE in the meta-enforcement step. Thus, provided we are in a state where at least one CE is present, the probability that a DE enters bad standing in a any given round is bounded above zero. It follows that for any $\bar{\beta} < 1$ and $\varepsilon < 1$ there is some round $\bar{t}(\varepsilon, \bar{\beta}) \geq 1$ such that if $t > \bar{t}(\varepsilon, \bar{\beta})$, then $\Pr\left(\beta > \bar{\beta}\right) > 1 - \varepsilon$. That is, for any round after $\bar{t}(\varepsilon, \bar{\beta})$, the fraction of the DE that are in bad standing is at least $\bar{\beta}$ with probability at least $1 - \varepsilon$.

Let T be the realised length of the repeated game. For any ε and $\bar{T}$ there is some $\bar{\delta}(\bar{T}, \varepsilon) < 1$ such that if $\delta > \bar{\delta}(\bar{T}, \varepsilon)$ then $\Pr\left(T > \bar{T}\right) > 1 - \varepsilon$. That is, the period lasts at least $\bar{T}$ rounds with probability at least $1 - \varepsilon$.

Now, fix $\bar{\beta}$ and ε, and pick any $\bar{T} > \bar{t}(\varepsilon, \bar{\beta})$. By making δ large enough we can ensure that $\Pr(T > \bar{T})$ is high enough. More precisely, if $\delta > \bar{\delta}(\bar{T}, \varepsilon)$, then with a probability of at least $(1 - \varepsilon)$ we have $T > \bar{T} > \bar{t}(\varepsilon, \bar{\beta})$. This implies that with a probability of at least $(1 - \varepsilon)^2$ the fraction of rounds in which $\beta > \bar{\beta}$ is at least $\left(\bar{T} - \bar{t}(\varepsilon, \bar{\beta})\right)/\bar{T}$. This shows that $\delta \to 1$ implies that $\left(\bar{T} - \bar{t}(\varepsilon, \bar{\beta})\right)/\bar{T} \to 1$. Furthermore, this holds for $\bar{\beta}$ arbitrarily close to 1.

Once a DE agent is in bad standing, she will remain there for an arbitrarily long time, since $\kappa \to \infty$. Hence, the average payoffs will be approximately equal to the payoff obtained when all CE are in good standing and all DE are in bad standing.

C.4. Meta-Enforcement. First consider states with $n_E \geq 2$, and $n_P \geq 2$. For the CE-type

$$\begin{aligned}\pi_{CE} = &\underbrace{\frac{n_{DE}}{n_E - 1}}_{\text{Probability of DE}} \left(\pi_{CE(E)} - l\right) + \underbrace{\frac{n_{CE} - 1}{n_E - 1}}_{\text{Probability of CE}} \pi_{CE(E)} \\ &= \pi_{CE(E)} - \frac{n_{DE}}{n_E - 1} l \\ &= \frac{n_P}{n_E}\tau\left(w + (b - c)\frac{n_{CP}}{n_P}\right) - v\frac{n_{DP}}{n_E} - f - \frac{n_{DE}}{n_E - 1} l\end{aligned}$$

and for the DE-type

$$\begin{aligned}\pi_{DE} = &\underbrace{\frac{n_{DE} - 1}{n_E - 1}}_{\text{Probability of DE}} \left(\pi_{DE(E)} - l\right) + \underbrace{\frac{n_{CE}}{n_E - 1}}_{\text{Probability of CE}} \left(\pi_{DE(E)} - l\right) \\ &= \pi_{DE(E)} - l \\ &= \frac{n_P}{n_E}\tau\left(w + (b - c)\frac{n_{CP}}{n_P}\right) - l.\end{aligned}$$

Now let us consider states where $n_E < 2$ or $n_P < 2$. If $n_E = 1$ (implying $n_P \geq 1$) then $\pi_{CE} = \pi_{CE(E)}$ and $\pi_{DE} = \pi_{DE(E)}$. If $n_P \leq 1$ (implying $n_E \geq 2$) then

$$\pi_{CE} = \pi_{CE(E)} - \frac{n_{DE}}{n_E - 1} l - f,$$

and

$$\pi_{DE} = \pi_{DE(E)} - l.$$

D. (Exact) Best-Reply Learning. As mentioned above, we consider a three-speed myopic best response process where agents may switch strategy within professions more often than they may switch profession (move between producer and enforcer strategies). The main idea behind this assumption is that it takes more time to learn a new trade than it takes to change one's behaviour given the current occupation.

At the beginning of each of the first n_P periods one producer is drawn at random (without replacement) and may choose whether to become a different kind of producer. When such an opportunity arises she chooses the strategy that would have maximised her previous per-round payoff. More formally, in period t player i chooses a strategy

$$s_i^t \in \arg \max_{s \in \{CP, DP\}} \pi(s, n^{t-1}),$$

where n^{t-1} is the state of the population (distribution of strategies) in period $t - 1$.

Once all producers have decided on their strategy one enforcer is drawn at random and may decide whether to become an enforcer of the other type. She is assumed to choose a myopic best response to the distribution of play in the previous period, i.e. she chooses

$$s_i^t \in \arg \max_{s \in \{CE, DE\}} \pi(s, n^{t-1}).$$

After this, all enforcers again receive the opportunity to update their strategy. Once all enforcers have updated their strategy, and all producers have had the opportunity to update following the last producer, one player is selected at random and may choose her strategy from the full set of strategies, i.e., in addition to the choice of cooperating or defecting she may decide on her profession. More formally, she chooses

$$s_i^t \in \arg \max_{s \in S} \pi(s, n^{t-1}).$$

Under the best response process agents take into account that their strategy choice will have an impact on the distribution of strategies in the overall population. In order to represent this we introduce the following notation. Given an agent with current strategy s who switches to s' and an initial population profile n, we denote the resulting population profile by $n^{s|s'}$, with $n_i^{s|s'} = n_i$ for all $i \neq s, s'$, $n_s^{s|s'} = n_s - 1$, and $n_{s'}^{s|s'} = n_{s'} + 1$. With this notation an agent with current strategy s will switch to another strategy s' with positive probability if

$$\max_{s' \in S} \pi(s'^{s|s'}) \geq \pi(s, n),$$

will switch with certainty if the above inequality holds strictly, and does not switch if it does not hold. In the case that producers and enforcers may only decide on their type, we have $S = \{CP, DP\}$ and $S = \{CE, DE\}$, respectively. If an agent may decide on the entire set of strategies we have $S = \{CP, DP, CE, DE\}$.

E. Analysis of the Unperturbed Dynamic. In a first step we will examine the circumstances under which producers will switch between cooperation and defection, when $n_P \geq 2$ and $n_E \geq 1$. A defecting producer will switch if $\pi(CP, n^{DP|CP}) \geq \pi(DP, n)$. Rewriting this condition reveals that a defecting producer will become a cooperator with positive probability if

$$n_{CE} \geq \frac{(1-\tau)c}{p} n_E,$$

will switch with certainty in case the inequality is strict, and will not switch in case it is violated. Likewise, a cooperating producer will become a defector with positive probability if $\pi(DP, n^{CP|DP}) \geq \pi(CP, n)$. She will stay in case this inequality is violated and will switch in case it does not hold. A cooperating producer will switch with positive probability whenever

$$n_{CE} \leq \frac{(1-\tau)c}{p} n_E.$$

Now consider enforcers. A defection enforcer will switch to cooperation enforcement with positive probability if $\pi(CE, n^{DE|CE}) \geq \pi(DE, n)$ or if

$$n_{CE} \geq \frac{f}{l}(n_E - 1).$$

She will switch with probability one in case of a strict inequality, and will stay in case the above inequality fails to holds. Similarly, a cooperation enforcer will switch with positive probability if $\pi(DE, n^{CE|DE}) \geq \pi(CE, n)$, will switch with certainty in case of a strict inequality and will remain a cooperation enforcer otherwise. This condition can be rewritten as

$$n_{CE} \leq \frac{f}{l}(n_E - 1) + 1.$$

Intuitively, players who consider switching will have one more co-player of the same type as players who remain at their strategy. Thus, if players decide to stay at their current action players with the other strategy will have to switch.

If the population is of size N then the set of states is

$$\mathcal{N} = \left\{ n \in \mathbb{N}^4 : n_{CP} + n_{DP} + n_{CE} + n_{DE} = N \right\}.$$

We define

$$E^C = \{n \in \mathcal{N} | n_{CP} = n_P, n_{CE} = n_E \text{ and } \alpha^C N - 1 \leq n_{CE} \leq \alpha^C N\},$$
$$E^D = \{n \in \mathcal{N} | n_{DP} = n_P, n_{DE} = n_E \text{ and } \alpha^D N - 1 \leq n_{DE} \leq \alpha^D N\},$$

where

$$\alpha^C = \frac{\tau(w+b-c)}{w+b+f-c} \quad \text{and} \quad \alpha^D = \frac{w\tau}{w+l}.$$

In the next lemma it will be shown that E^C and E^D correspond to the cooperative and the defecting absorbing sets of our dynamic process. In both sets there are only enforcers and producers of one type present in the population and the relative size of the two groups is determined by the fundamentals of the model.

Lemma S1 *Consider states where there is at least two enforcers and at least two producers are present in the population ($n_E \geq 2$ and $n_P \geq 2$). If $p > (1-\tau)c$ and if N is sufficiently large, the following holds:*

1. *From any state with $n_{CE} > \frac{f}{l}(n_E - 1) + 1$ the process converges to E^C with probability one,*
2. *from any state with $n_{CE} < \frac{f}{l}(n_E - 1)$ the process converges to E^D with probability one, and*
3. *from any state with $\frac{n_{CE}-1}{n_E-1} \leq \frac{f}{l} \leq \frac{n_{CE}}{n_E-1}$ the process converges to either E^C or E^D with positive probability.*

Proof. *First*, consider states with $n_{CE} > \frac{f}{l}(n_E - 1) + 1$. After all producers have updated their strategy an enforcer is drawn to decide whether to become an enforcer of the other type. For $n_{CE} > \frac{f}{l}(n_E - 1) + 1$ enforcers who are currently cooperative will stay and defecting enforcers will become cooperation enforcers. Thus, with probability one the process converges to a state where $n_{CE} = n_E$. As $\frac{n_{CE}}{n_E} = 1 > \frac{(1-\tau)c}{p}$ (due to the assumption that $p > (1-\tau)c$), all producers will either switch to become cooperative producers or remain cooperative producers. We have reached a state with $n_{CE} = n_E$ and $n_{CP} = n_P$.

Now consider the stage where agents may choose among all four different strategies (at a state with $n_{CE} = n_E$ and $n_{CP} = n_P$). If a cooperation enforcer is drawn, she will not decide to become a defecting producer since $\frac{n_{CE}}{n_E} = 1$ implies that a cooperative producer will always earn a higher payoff. Likewise, she will not decide to become a defection enforcer since cooperation enforcers earn more. Thus, a cooperation enforcer may either decide to stay a cooperation enforcer or become cooperative producer. She will switch strategies if $\pi(CP, n^{CE|CP}) > \pi(CE, n)$, will remain if $\pi(CP, n^{CE|CP}) < \pi(CE, n)$, and will randomise between the two options if $\pi(CP, n^{CE|CP}) = \pi(CE, n)$. One can check that a cooperation enforcer will switch with certainty if

$$n_{CE} > \alpha^C N.$$

(Note that the system moves towards the rest point away from the vertices, so the system remains in the set of states where $n_E \geq 2$ and $n_P \geq 2$.)

If a cooperative producer is drawn she will never decide to become a defecting producer or a defection enforcer (since $\frac{n_{CE}}{n_E} = 1$). A cooperative producer will become a cooperation enforcer with certainty if $\pi(CE, n^{CP|CE}) > \pi(CP, n)$. First, consider the case where after the switch there are at least two producers left, $n_{CP} = n_P \geq 3$. Note that in this case $\pi(CE, n^{CP|CE}) = \frac{n_P - 1}{n_E + 1}\tau(w - b - c) - f > (1 - \tau)(w + b - c) = \pi(CP, n)$ is equivalent to

$$n_{CE} < \alpha^C N - 1, \tag{S7}$$

Now, consider the special case where after the switch there is only one producer left, $n_{CP} = n_P = 2$. Since this lone producer receives less payoff also the income of the switching enforcers changes to $\pi(CE, n^{CP|CE}) = \frac{n_P - 1}{n_E + 1}\tau w - f$ and we have that a producer becomes an enforcer with certainty whenever $\pi(CE, n^{CP|CE}) = \frac{n_P - 1}{n_E + 1}\tau w - f > \pi(CP, n) = (1 - \tau)(w + b - c)$ which reduces to

$$N < 1 + \frac{\tau w}{f + (1 - \tau)(w + b - c)}. \tag{S8}$$

Thus, provided the population is sufficiently large (so that the previous inequality is violated) cooperating producers will never choose to become cooperating enforcers if that would leave only one producer left.

Thus, for all states where $n_{CE} > \alpha^C N$ the cooperation enforcers will switch and the cooperative producers will stay, thus reducing the number of enforcers. Similarly, for $n_{CE} < \alpha^C N - 1$ enforcers will stay and producers will switch, increasing the number of enforcers.

Now let us consider states with $\alpha^C N - 1 \leq n_{CE} \leq \alpha^C N$. If $\alpha^C N \notin \mathbb{Z}$, both, enforcers and producers will stay with probability one, implying that the state $n_{CE} = \lfloor \alpha^C N \rfloor$ constitutes a singleton absorbing set. If $\alpha^C N \in \mathbb{Z}$ and $n_{CE} = \alpha^C N - 1$ the enforcers will stay with certainty and the producers will randomise. Thus, we might move to a state with $n_{CE} = \alpha^C N$. At this state producers will stay with probability one and the enforcers will randomise, thus moving us back to a state with $n_{CE} = \alpha^C N - 1$ with positive probability. Hence, in the (non-generic) case $\alpha^C N \in \mathbb{Z}$ there exists a non-singleton absorbing set.

Finally, we need to check that in these absorbing sets there are at least two producers and at least two enforcers. First consider $\alpha^C N \notin \mathbb{Z}$. To make sure that there are at least two enforcers, we require $n_{CE} = \lfloor \alpha^C N \rfloor \geq 2$ which translates into $N \geq \frac{2}{\alpha^C}$. To ensure there are at least two producers, we need to have $n_{CE} = \lfloor \alpha^C N \rfloor \leq N - 2$ which holds if $N > \frac{1}{1 - \alpha^C}$. If $\alpha^C N \in \mathbb{Z}$ the number of enforcers fluctuates between $\alpha^C N$ and $\alpha^C N - 1$. If $N \geq \frac{1}{\alpha^C}$ there are always two enforcers ($\alpha^C N - 1 \geq 2$) and if $N \geq \frac{2}{1 - \alpha^C}$ there are at least two producers ($\alpha^C N \leq N - 2$). Provided, the population is sufficiently large, the previous four inequalities hold. Finally note that for N sufficiently large also inequality S8 will be violated so that cooperating producers will never choose to become cooperating enforcers if this would leave only one producer left.

Consider now the *second* case, $n_{CE} < \frac{f}{l}(n_E - 1)$. An argument akin to the one used above shows that the process converges to a state where $n_{CE} = n_E \geq 2$ and $n_{CP} = n_P \geq 2$ when agents can only decide on their behaviour within professions. Further, as above, defecting producers and defecting enforcers will never become cooperative producers or enforcers. A defection enforcer will switch strategies with probability one if $\pi(DP, n^{DE|DP}) > \pi(DE, n)$, which can be rewritten as

$$n_{DE} > \alpha^D N.$$

(Note that the system moves towards the rest point and away from the boundary, so the system remains in the set of states where $n_E \geq 2$ and $n_P \geq 2$.) Likewise, a defecting producer will become a defection enforcer with certainty if $\pi(DE, n^{DP|DE}) > \pi(DP, n)$, or

$$n_{DE} < \alpha^D N - 1.$$

As above, if $\alpha^D N \notin \mathbb{Z}$ the state $n_{DE} = \lfloor \alpha^C N \rfloor$ corresponds to the (singleton) absorbing set. If $\alpha^D N \in \mathbb{Z}$ the absorbing set contains the two states $\alpha^D N - 1$ and $\alpha^D N$.

Again, we need to make sure that at the absorbing set there are at least two enforcers and at least two producers. For $\alpha^D N \notin \mathbb{Z}$ this is the case if $N \geq \frac{2}{\alpha^D}$ and $N > \frac{1}{1 - \alpha^D}$. For $\alpha^D N \notin \mathbb{Z}$ this is true for $N \geq \frac{1}{\alpha^D}$ and $N \geq \frac{2}{1 - \alpha^D}$. Note that all of this inequalities hold for N sufficiently large.

Finally, consider the case $\frac{f}{l}(n_E - 1) \leq n_{CE} \leq \frac{f}{l}(n_E - 1) + 1$. First, consider the case where $\frac{f}{l}(n_E - 1) \notin \mathbb{Z}$. If an cooperation enforcer is first drawn, she will switch to become a defection enforcer, implying that we move to $n_{CE} - 1 < \frac{f}{l} n_E - 1$. Thus, we will eventually converge to E^D. Conversely, if a defection enforcer is first drawn she will switch to become a cooperation enforcer, implying that we eventually converge to E^D.

Now consider $\frac{f}{l}(n_E - 1) \in \mathbb{Z}$. If $n_{CE} = \frac{f}{l}(n_E - 1)$, a revising cooperation enforcer will switch and a revising defection enforcer will randomise. If the former agent is drawn to revise, we end up in E^D. If the latter is drawn we may end up in either E^C or E^D. Likewise, if $n_{CE} = \frac{f}{l}(n_E - 1)$ the process may either end up in E^C or E^D, depending on who is drawn to revise and how an indifferent cooperation enforcer decides. ■

If $f > l$ then $n_{CE} < \frac{f}{l}(n_E - 1) + 1$ so the above lemma implies that process converges to E^D with probability one. In this case the analysis is trivial, so in what follows we assume that $f < l$. In a similar vein if $p < (1 - \tau) c$ then it is easy to see that CP will be outperformed by DP at all states. Thus we focus on the case $p < (1 - \tau) c$.

The next lemma characterises the dynamic process in the absence of enforcers, $n_E = 0$, and when there is only one enforcer, $n_E = 1$.

Lemma S2 *Consider states with* $n_E < 2$. *If* $p > (1-\tau)\, c$, *and if* N *is large enough, then the process converges to* E^D *with probability one.*

Proof. Suppose $n_E = 0$, so $n_P = N$. First, note that in the absence of enforcers we will move to a state where all producers defect since

$$\pi\left(DP, n^{CP|DP}\right) = w + \frac{n_{CP}-1}{n_P-1}b > w - c + \frac{n_{CP}-1}{n_P-1}b = \pi\left(CP, n\right)$$

and

$$\pi\left(CP, n^{DP|CP}\right) = w - c + \frac{n_{CP}}{n_P-1}b < w + \frac{n_{CP}}{n_P-1}b = \pi\left(DP, n\right).$$

Thus, we will move to a state where there are only defecting producers, $n_{DP} = N$, and each will earn a payoff of w. Now consider the stage where producers may decide among the full set of strategies. In this case we have $\pi\left(DE, n^{DP|DE}\right) = \tau\left(N-1\right)w$ and $\pi\left(CE, n^{CP|CE}\right) = \tau\left(N-1\right)w - f$. (Note, we do not subtract l from DE's payoff since there is only one DE in the population.)

By assumption $\tau > 1/\left(N-1\right)$. It follows that

$$\pi\left(DE, n^{DP|DE}\right) = \tau\left(N-1\right)w > w = \pi\left(DP, n\right).$$

Thus, if a producer is drawn to chose a strategy from the full set of strategies she chooses to become DE. Thus the system moves to a state with $n_E = n_{DE} = 1$.

Suppose $n_E = n_{DE} = 1$. All producers chose to become DP because, for N large enough so that $\frac{1}{N-1}b < c$, it holds that

$$\pi\left(CP, n\right) = \left(1-\tau\right)\left(w - c + \frac{n_{CP}}{n_P}b\right) < \left(1-\tau\right)\left(w + \frac{n_{CP}-1}{n_P}b\right) = \pi\left(DP, n^{CP|DP}\right)$$

and

$$\pi\left(CP, n^{DP|CP}\right) = \left(1-\tau\right)\left(w - c + \frac{n_{CP}+1}{n_P}b\right) < \left(1-\tau\right)\left(w + \frac{n_{CP}}{n_P}b\right) = \pi\left(DP, n\right).$$

Once all producers are DP, the enforcer gets to choose between DE and CE, and clearly prefers DE, for the same reasons as above. Next someone (one of the producers or the only enforcer) is allowed to choose among all four strategies. If it is the only enforcer that gets this choice she chooses to remain DE. What happens if one of the producers is allowed to choose among all four strategies? The state n is such that $n_E = n_{DE} = 1$ and $n_P = n_{DP} = N-1$. We have

$$\begin{aligned}
\pi\left(DP, n\right) &= \left(1-\tau\right)w, \\
\pi\left(DE, n^{DP|DE}\right) &= \frac{N-2}{2}\tau w - l, \\
\pi\left(CE, n^{DP|CE}\right) &= \frac{N-2}{2}\tau w - l - f.
\end{aligned}$$

(Note that if there is one CE and one DE then they will fight in the meta-enforcement stage.) We have $\pi\left(DE, n^{DP|DE}\right) > \pi\left(CE, n^{DP|CE}\right)$, and if N is large enough ($N > 2\frac{w+l}{\tau w}$) then we have $\pi\left(DE, n^{DP|DE}\right) > \pi\left(DP, n\right)$. Thus a producer chooses to become DE. We arrive at a state with $n_E = n_{DE} = 2$ and $n_P = n_{DP} = N-2$. From the previous lemma we know that this state is in the basin of attraction for E^D.

Suppose $n_E = n_{CE} = 1$. What do producers do?

$$\begin{aligned}
\pi\left(CP, n\right) &= \left(1-\tau\right)\left(w - c + \frac{n_{CP}}{n_P}b\right), \\
\pi\left(CP, n^{DP|CP}\right) &= \left(1-\tau\right)\left(w - c + \frac{n_{CP}+1}{n_P}b\right), \\
\pi\left(DP, n\right) &= \left(1-\tau\right)\left(w + \frac{n_{CP}}{n_P}b\right) - p, \\
\pi\left(DP, n^{CP|DP}\right) &= \left(1-\tau\right)\left(w + \frac{n_{CP}-1}{n_P}b\right) - p.
\end{aligned}$$

We have $\pi\left(CP, n\right) > \pi\left(DP, n^{CP|DP}\right)$ and $\pi\left(CP, n^{DP|CP}\right) > \pi\left(DP, n\right)$ if $p > \left(1-\tau\right)\left(c - \frac{1}{n_P}b\right)$, which is implied by the assumption that $p > \left(1-\tau\right)c$. Thus all producers become CP, so we are at a state where $n_E = n_{CE} = 1$ and $n_P = n_{CP} = N-1$. Next the only enforcer chooses between CE and DE. Since

$$\pi\left(DE, n^{CE|DE}\right) = \frac{N-1}{1}\tau\left(w + b - c\right) > \frac{N-1}{1}\tau\left(w + b - c\right) - f = \pi\left(CE, n\right),$$

she chooses to become DE, so we are at a state where $n_E = n_{DE} = 1$ and $n_P = n_{CP} = N-1$. Next all producers get to adjust and they all move from CP to DP, as explained above (assuming N is large enough so that $b \leq c\left(N-1\right)$). Following the above reasoning (for the case of $n_E = n_{DE} = 1$ and $n_P = n_{DP} = N-1$) we arrive at a state with $n_E = n_{DE} = 2$ and $n_P = n_{DP} = N-2$, and from the previous lemma we know that this state is in the basin of attraction for E^D. ∎

The next lemma characterises the dynamic process in the absence of producers, $n_P = 0$, and when there is only one producer, $n_P = 1$.

Lemma S3 *Consider states with $n_P < 2$. If $p > (1-\tau)\,c$, and if N is large enough, the following holds:*

1. *From any state with $n_{CE} > \frac{f}{l}(n_E - 1) + 1$ the process converges to E^C with probability one,*
2. *from any state with $n_{CE} < \frac{f}{l}(n_E - 1)$ the process converges to E^D with probability one, and*
3. *from any state with $\frac{n_{CE}-1}{n_E-1} \leq \frac{f}{l} \leq \frac{n_{CE}}{n_E-1}$ the process converges to either E^C or E^D with positive probability.*

Proof. Suppose $n_P = 0$. We have

$$\pi\,(CE, n) = -\frac{n_{DE}}{n_E - 1} l - f,$$
$$\pi\left(CE, n^{DE|CE}\right) = -\frac{n_{DE} - 1}{n_E - 1} l - f,$$
$$\pi\,(DE, n) = \pi\left(DE, n^{CE|DE}\right) = -l.$$

A cooperation enforcer will switch with positive probability if $\pi(DE, n^{CE|DE}) \geq \pi(CE, n)$, or equivalently

$$n_{CE} \leq \frac{f}{l}(n_E - 1) + 1.$$

A defection enforcer will switch with positive probability if $\pi(CE, n^{DE|CE}) \geq \pi(DE, n)$, or equivalently

$$n_{CE} \geq \frac{f}{l}(n_E - 1).$$

These are the same conditions as when $n_E \geq 2$ and $n_P \geq 2$. Next when enforcers are allowed to choose among all strategies

$$\pi\left(CP, n^{CE|CP}\right) = \pi\left(DP, n^{CE|DP}\right) = \pi\left(CP, n^{DE|CP}\right) = \pi\left(DP, n^{DE|DP}\right) = (1-\tau)\,w,$$

so a revising enforcer chooses to become a producer, and chooses both CP and DP with positive probability.

Suppose $n_P = 1$. The unique producer is indifferent between CP and DP. When an enforcer is allowed to chose between enforcer strategies we have

$$\pi\,(CE, n) = \frac{1}{N-1}\tau w - \frac{n_{DE}}{n_E - 1} l - f,$$
$$\pi\left(CE, n^{DE|CE}\right) = \frac{1}{N-1}\tau w - \frac{n_{DE} - 1}{n_E - 1} l - f,$$
$$\pi\,(DE, n) = \pi\left(DE, n^{CE|DE}\right) = \frac{1}{N-1}\tau w - l.$$

Again, cooperation enforcer will switch with positive probability if

$$n_{CE} \leq \frac{f}{l}(n_E - 1) + 1$$

and a defection enforcer will switch with positive probability if

$$n_{CE} \geq \frac{f}{l}(n_E - 1).$$

Thus, when enforcers are allowed to choose among all strategies we are at either of four different states: (i) $n_E = n_{CE} = N - 1$ and $n_P = n_{CP} = 1$ (ii) $n_E = n_{CE} = N - 1$ and $n_P = n_{DP} = 1$, (iii) $n_E = n_{DE} = N - 1$ and $n_P = n_{CP} = 1$, or (iv) $n_E = n_{DE} = N - 1$ and $n_P = n_{DP} = 1$.

In case (i) we have

$$\pi\left(CP, n^{DE|CP}\right) = \pi\left(CP, n^{CE|CP}\right) = (1-\tau)\,(w - c + b)$$

and

$$\pi\left(DP, n^{DE|DP}\right) = \pi\left(DP, n^{CE|DP}\right) = (1-\tau)\,(w + b) - p.$$

In case (ii) we have

$$\pi\left(CP, n^{DE|CP}\right) = \pi\left(CP, n^{CE|CP}\right) = (1-\tau)\,(w - c)$$

and

$$\pi\left(DP, n^{DE|DP}\right) = \pi\left(DP, n^{CE|DP}\right) = (1-\tau)\,w - p.$$

In either case the payoffs to CP are strictly larger than the payoffs to DP, by the assumption that $p > (1-\tau)\,c$. Moreover the payoff to CP is larger than what is obtained by remaining an enforcer, provided that N is large enough ($N > \frac{\tau w}{(1-\tau)(w-c)+f} + 1$). Thus, we arrive at a state with $n_P = 2$ and $n_E = n_{CE} = N - 2$. From the first lemma we know that this is in the basin of attraction of E^C.

In case (iii) we have

$$\pi\left(CP, n^{DE|CP}\right) = \pi\left(CP, n^{CE|CP}\right) = (1-\tau)(w-c+b)$$

and

$$\pi\left(DP, n^{DE|DP}\right) = \pi\left(DP, n^{CE|DP}\right) = (1-\tau)(w+b).$$

In case (iv) we have

$$\pi\left(CP, n^{DE|CP}\right) = \pi\left(CP, n^{CE|CP}\right) = (1-\tau)(w-c)$$

and

$$\pi\left(DP, n^{DE|DP}\right) = \pi\left(DP, n^{CE|DP}\right) = (1-\tau)w.$$

In either case the payoffs to DP are strictly larger than the payoffs to CP. Moreover, the payoff to DP is larger than what is obtained be remaining an enforcer, provided that N is large enough ($N > \frac{\tau w}{(1-\tau)w+l} + 1$). Thus, we arrive at a state with $n_P = 2$ and $n_E = n_{DE} = N-2$. From the first lemma we know that this is in the basin of attraction of E^D. ■

Observation S1 *Under the maintained assumption that $f < l$, it is always the case that $\alpha^C > \alpha^D$. Moreover, as $N \to \infty$, the payoff in the cooperation equilibrium is*

$$\pi^C = (w+b-c)\left(1-\alpha^C\right) - f\alpha^C = (1-\tau)(w+b-c)$$

and payoff in the defection equilibrium is

$$\pi^D = w\left(1-\alpha^D\right) - l\alpha^D = (1-\tau)w.$$

Payoff in the absence of enforcers (where all producers defect) is

$$\pi^P = w.$$

Note that we always have $\pi^P > \pi^D$ (since $\tau > 0$) and $\pi^C > \pi^D$ (since $b > c$). Finally $\pi^C > \pi^P$ if and only if $(1-\tau)(w+b-c) > w$.

F. Analysis of the Perturbed Dynamic: Stochastic Stability. The following theorem characterises the set of stochastically stable states.

Lemma S4 *Suppose N is large enough, $p > (1-\tau)c$, and $f < l$. The radius and coradius of the defection equilibrium E^D and cooperation equilibrium E^C satisfy*

$$CR(E^C) = R(E^D) = \left\lceil \frac{f}{l}\left\lceil \alpha^D N - 2\right\rceil \right\rceil$$

and

$$CR(E^D) = R(E^C) = \left\lceil \left(1-\frac{f}{l}\right)\left\lceil \alpha^C N - 2\right\rceil \right\rceil.$$

Proof. First, consider the defection equilibrium, E^D with $n_{CE} = 0$. There is only one way to move into the basin of attraction of E^C, namely to move to a state where $n_{CE} \geq \frac{f}{l}(n_E - 1)$ or equivalently $\frac{n_{CE}}{n_E-1} \geq \frac{f}{l}$ (lemma S1 and lemma S3). We want to move from a state where $\frac{n_{CE}}{n_E-1} = 0$ to a state where $\frac{n_{CE}}{n_E-1} \geq \frac{f}{l}$. Mutations may either change the number n_{CE}, n_E or both. We are looking for the least costly way of increasing the fraction $\frac{n_{CE}}{n_E-1}$. If one defection enforcer mutates and becomes a cooperation enforcer we move to a new state which is characterised by $\frac{n_{CE}+1}{n_E-1}$. If a defection enforcer mutates to become a producer we move to a state with $\frac{n_{CE}}{n_E-2}$. If a producer switches to become a cooperation enforcer we move to a state $\frac{n_{CE}+1}{n_E}$. Note that we have $\frac{n_{CE}+1}{n_E} < \frac{n_{CE}+1}{n_E-1}$. Thus, it is more cost-effective to switch defection enforcers to cooperation enforcers than to switch producers to cooperation enforcers. Further, we have $\frac{n_{CE}+1}{n_E-1} \geq \frac{n_{CE}}{n_E-2}$ if $n_E \geq n_{CE}+2$. Thus, up until the point where all but one enforcers are cooperative, $n_{CE} = n_E - 1$, a mutation from an defection enforcer to a cooperation enforcer increases the fraction $\frac{n_{CE}}{n_E-1}$ more than any other mutation could. Note that at the point $n_{CE} = n_E - 1$ we have that the remaining defection enforcer will switch with certainty, since $n_{CE} - 1 > \frac{f}{l}(n_E-1)$. Thus, when calculating the number of mutations required for a transition we only need to consider mutations from defection enforcers to cooperation enforcers.

To move out of the basin of attraction with positive probability we need $n_{CE} \geq \frac{f}{l}(n_E - 1)$. Let m^{DC} be the number of mutations required to move from E^D to E^C, via increasing $\frac{n_{CE}+1}{n_E-1}$ (lemma S1), with positive probability. Note that at the absorbing set E^D the number of enforcers is given by $\alpha^D N - 1 \leq n^{DE} \leq \alpha^D N$. If $\alpha^D N \notin \mathbb{Z}$ we have $n^{DE} = \lceil \alpha^D N \rceil - 1$ and consequently $m^{DC} = \left\lceil \frac{f}{l}\lceil \alpha^D N - 2\rceil\right\rceil$. If $\alpha^D N \in \mathbb{Z}$ we fluctuate between states with $\alpha^D N - 1$ and $\alpha^D N$ enforcers. If the mutations happen when the number of enforcers is at its lowest we have $m^{DC} = \left\lceil \frac{f}{l}\left(\alpha^D N - 2\right)\right\rceil$. Consequently, regardless of whether $\alpha^D N \in \mathbb{Z}$ or not $m^{DC} = \left\lceil \frac{f}{l}\left\lceil \alpha^D N - 2\right\rceil\right\rceil$.

Now consider the cooperation equilibrium, E^C with $n_{CE} = n_E$. There are two possible ways to escape its basin of attraction: (i) move to a state where $n_{CE} \leq \frac{f}{l}(n_E - 1) + 1$ or equivalently $\frac{n_{CE}-1}{n_E-1} \leq \frac{f}{l}$ (lemma S1) or (ii) move to a state with $n^E \leq 1$ enforcers (lemma S2). First consider the former alternative. If a cooperation enforcer switches to become a defection enforcer we move to a state with $\frac{n_{CE}-2}{n_E-1}$, if a cooperation enforcer becomes a producer we move to a state with $\frac{n_{CE}-2}{n_E-2}$, if a

producer becomes a defection enforcer we reach a state with $\frac{n_{CE}-1}{n_E}$.* Clearly, the first effect dominates the second. Further, $\frac{n_{CE}-2}{n_E-1} \le \frac{n_{CE}-1}{n_E-2}$ holds whenever $n_{CE} \le n_E - 1$. Thus, the most effective way to decrease $\frac{n_{CE}}{n_E-1}$ is by turning cooperation enforcers in defection enforcers. Let m^{CD} be the number of required mutations to move out of the basin of attraction of E^C with positive probability via decreasing the number of cooperation enforcers to $n_{CE} \le \frac{f}{l}(n_E - 1) + 1$, (lemma S1). This requires $\lceil n_E - 1 - \frac{f}{l}(n_E - 1) \rceil$ cooperation enforcers to become defection enforcers. Currently, we have $n_{CE} = n_E$ cooperation enforcers with $\alpha^C N - 1 \le n_{CE} \le \alpha^C N$. If $\alpha^C N \notin \mathbb{Z}$, the minimal number of mutations to enter the defection equilibrium E^D is characterised by $m^{CD} = \left\lceil \left(1 - \frac{f}{l}\right) \lceil \alpha^C N - 2 \rceil \right\rceil$. If $\alpha^C N \in \mathbb{Z}$ the number of enforcers fluctuates between $\alpha^C N - 1$ and $\alpha^C N$. We pick the state where the number of enforcers is minimal at $\alpha^C N - 1$. Thus we require $m^{CD} = \left\lceil \left(1 - \frac{f}{l}\right) \left(\alpha^C N - 2\right) \right\rceil$ mutations in this case. Again, we can write the number of required mutations as $m^{CD} = \left\lceil \left(1 - \frac{f}{l}\right) \lceil \alpha^C N - 2 \rceil \right\rceil$ (regardless of whether $\alpha^C N \in \mathbb{Z}$ or not).

The alternative way to escape the basin of attraction of E^C is to move to a state with $n^E \le 1$ (lemma S2). This requires $n^{CE} - 1$ mutations. Thus, moving out of E^C with positive probability requires $\tilde{m}^{CD} = \lceil \alpha^C N - 1 \rceil$ mutations. Since $(1 - \frac{f}{l}) < 1$, it is more cost-effective to change cooperation enforcers into defection enforcers than to reduce the number of producers to one, $m^{CD} \le \tilde{m}^{CD}$. It follows that $R(E^C) = CR(E^D) = m^{CD}$. ■

We now identify conditions under which either of the two equilibria is stochastically stable.

Theorem S2 *Suppose $p > (1 - \tau)\, c$, $f < l$, and $\alpha^D < 1/2$. In a sufficiency large population, if*

$$\left(\frac{l}{f} - 1\right)\left(1 + \frac{l}{w}\right) > \left(1 + \frac{f}{w + b - c}\right),$$

then the cooperation equilibrium E^C is stochastically stable ($R(E^C) > CR(E^C)$), and if

$$\left(\frac{l}{f} - 1\right)\left(1 + \frac{l}{w}\right) < \left(1 + \frac{f}{w + b - c}\right),$$

the defection equilibrium E^D is stochastically stable ($R(E^D) > CR(E^D)$).

Proof. In order for E^C to be stochastically stable we need $R(E^C) > CR(E^C)$. This holds if

$$\left\lceil \left(1 - \frac{f}{l}\right) \lceil \alpha^C N - 2 \rceil \right\rceil > \left\lceil \frac{f}{l} \lceil \alpha^D N - 1 \rceil \right\rceil.$$

This holds if

$$\left(1 - \frac{f}{l}\right) \lceil \alpha^C N - 2 \rceil > \frac{f}{l} \lceil \alpha^D N - 1 \rceil + 1.$$

Because $\lceil \alpha^C N \rceil \ge \alpha^C N$ and $\lceil \alpha^D N \rceil \le \alpha^D N + 1$ a sufficient condition for the previous inequality to hold is

$$\left(1 - \frac{f}{l}\right)\left(\alpha^C N - 2\right) > \frac{f}{l}\left(\alpha^D N\right) + 1.$$

This can be rewritten as

$$N\left(\alpha^C\left(1 - \frac{f}{l}\right) - \alpha^D \frac{f}{l}\right) > 3 - 2\frac{f}{l}.$$

If $\alpha^C\left(1 - \frac{f}{l}\right) > \alpha^D \frac{f}{l}$ the left hand side is linearly increasing in N while the right hand side is independent of N. It follows that there exists an N^* such that for all $N > N^*$ the above inequality holds and E^C is stochastically stable. A similar line of reasoning establishes that when $\alpha^C\left(1 - \frac{f}{l}\right) < \frac{f}{l}\alpha^D$ the state E^D is stochastically stable provided that the population is sufficiently large. ■

S3. Discussion of Other Ways of Obtaining Analytical Results Regarding the Invariant Distribution

There are a number of ways to make it possible to solve for the invariant distribution (apart from simulating the evolutionary process). When the number of strategies is small and the population is not too large, one can explicitly compute the transition matrix and then solve for the relevant eigenvector. However, in more complicated cases such as ours, some simplifying assumptions need to be imposed.

Recall that, with probability ε, a revising agent switches to another strategy that is drawn uniformly randomly. Such payoff-independent revisions are typically referred to as mutations, though in a model of social learning they should be interpreted as instances experimentation or mistakes, rather than genetic mutations. With probability $1 - \varepsilon$ the agent revises in a payoff-sensitive way by choosing another strategy with a probability that is increasing in the payoff the strategy would earn at the current population state (3). The probability by which the highest-earning strategies are chosen is governed by the noise parameter η. When η goes to zero the highest earning strategies are chosen with probability one and when η goes to

* If a producer becomes a cooperative enforcer we move to state with $(n_{CE} + 1)/n_E$. We have $(n_{CE} + 1)(n_E) \ge (n_{CE} - 1)/(n_E - 1)$ for $n^{CE} \le n^E - 1$. Thus, producers switching to become cooperative enforcers increase the fraction $(n_{CE} - 1)/(n_E - 1)$.

infinity all strategies are chosen with equal probability. In models of genetic evolution, the inverse of η is often referred to as the intensity of selection (5).

One common approach, which we follow, is to consider the limit of rare mutations, i.e. let ϵ vanish. In the case of imitative (or birth-death) processes it is particularly convenient to study the limit of rare mutations since the invariant distribution then puts almost all weight on a monomorphic state (6). While this approach is suitable when the equilibria of interest are monomorphic, our paper is concerned with the stability of mixed equilibria (i.e., polymorphic states), where a producer strategy and an enforcer strategy co-exist. One could attempt to overcome this difficulty by assuming that agents can be programmed to play mixed strategies (7), thereby making mixed equilibria correspond to monomorphic population states. However, this is not an attractive option in our setting, since it would imply that our cooperative equilibria are maintained *not* by the co-existence of enforcers and producers, but by the presence of a single type that mixes between being an enforcer and a producer. By contrast, in the case of a (smooth or exact) best-response dynamic like the one we use, mixed equilibria may correspond to polymorphic absorbing states.

Other approaches create scope for analytical solutions by taking the payoff sensitivity of revisions to some limit. By letting the payoff sensitivity of revisions go to zero (which in our model would imply taking the noise parameter η to infinity), one obtains what is known as the limit of weak selection (5). Typically, this assumption is invoked in the context of imitative processes or birth-death processes. One, then, proceeds by identifying the strategies whose share in the invariant distribution is above (favoured) or below (not favoured) their respective share in the uniform distribution (8). Again, the fact that we are interested in genuinely polymorphic mixed equilibria makes this approach unappealing. We are interested in whether a combination of strategies has a selective advantage together, not whether a single strategy has an advantage on its own (as in (7)). For this, instead of making revisions arbitrarily insensitive to payoff, we make them maximally sensitive to payoff by letting η go to zero so that the payoff-sensitive revisions become exact best responses.

It should be noted that our simulations approximate the invariant distribution without imposing any limiting assumptions on either mutation rates or the intensity of selection. The limiting assumptions are only made for analytical tractability. Nevertheless, we find coherence between our simulation results and the analytical results derived under the limiting assumptions.

S4. Numerical Approach

A. Simulation Methods. In order to approximate the invariant distribution we simulate the learning process for seven different (randomly drawn) initial conditions and compute the the time average of the different strategies over the seven runs. We iterate the repeated game over 10^6 periods (i.e. 10^6 instances of the repeated game and equally many revisions), with one agent revising in each iteration, in a population of 50 agents. Each round of the repeated game is played out as described in fig. 1 of the main text. We use the baseline parameter values reported in Table S1

In the case of an odd number of producers or an odd number of enforcers some agents will not be matched. In the production step unmatched producers (in case of an odd number of producers) simply earn the autarky payoff w. In the enforcement step unmatched enforcers do not earn any tax revenue and cannot punish any producers in the enforcement step. In the meta-enforcement step unmatched enforcers (in case of an odd number of enforcers) keep the payoff they earned in step 2, and their reputation is unaltered.

Revisions occur as described in the main text in the Dynamic Framework section. When evaluating payoffs for revisions, the various π_Y in eq. (6) of the main text are the realised average payoffs of Y-strategy agents in the current round.

B. Sensitivity to Parameters in the Presence of the PE strategy. Figure S2 shows the effect of adding PE to the set of strategies, under the same conditions as in fig. 4 of the main text. Overall the results are similar to those obtained in the absence of PE. Naturally, when the variable cost v is increased sufficiently, PE replaces CE, since the cost is only paid by the latter. For the very high levels of the continuation probability δ there is a sharp increase in PE and a corresponding drop in CE. We believe that this is due to the fact that a population of only DE and PE becomes an equilibrium when δ is sufficiently high (higher than what is needed for an equilibrium consisting of only DE and CE to exist).

C. Robustness to Noise. Figure S3 displays the results of varying action mistake probability μ, logit precision η, and revision mistakes ε. We consider both the case when the set of enforcer strategies consists of only CE and DE, and the one when PE is added to the mix. For modest levels of noise, the effect on cooperation is small. When action mistakes increase beyond 2.5% cooperation deteriorates substantially. We stress that this is the probability of single mistake. A typical enforcer faces four producers and one enforcer in each round, meaning that, in our baseline, the probability of making at least one mistake is approximately 12%. So, the typical CE has a 12% risk of acting in a way that confers bad standing.

D. Robustness to Initial Reputations. It is desirable that the reputation system is robust to mistakes. To this end, our simulations have included action (execution) mistakes, which ensure that all kinds of enforcers may end up in bad standing. As a further robustness check, we now consider starting our simulations at a state where all enforcers are in bad standing. The results are shown in Figure S4.

Figure S4a shows the results from simulations where enforcers begin with maximal bad standing (i.e. with bad standing equal to κ), for varying values of κ. Interestingly, this panel illustrates a trade-off. On the one hand, higher values of κ imply that CE have a harder time to shed off their bad reputation. On the other hand, higher values of κ make it harder for DEs to gain good standing (through mistakes or through being randomly matched to CPs in step 2 and enforcers in bad standing in

step 3) and consequently avoid punishment. This can be seen in panel Figure S4a, where maximal cooperation is reached for $\kappa = 2$.

Figure S4b shows that as enforcers start off with increasingly worse standing, the advantage conferred to the CE strategy decreases and cooperation decreases in the invariant distribution (numerically aproximated by time averages). This is to be expected. Note that, in our baseline, the expected length of a period (supergame) is 10 rounds and the number of punishment rounds is $\kappa = 8$. This means that if everyone starts out in bad standing, then for at least 4/5 of their expected lifespan both CE and DE agents will be in bad standing. So, there are very few rounds in which CEs can earn a higher payoff than DEs. With less extreme initial reputations, the CEs spend a smaller fraction of their expected lifespan in bad standing, hence they can—and eventually do—earn higher payoffs than DEs.

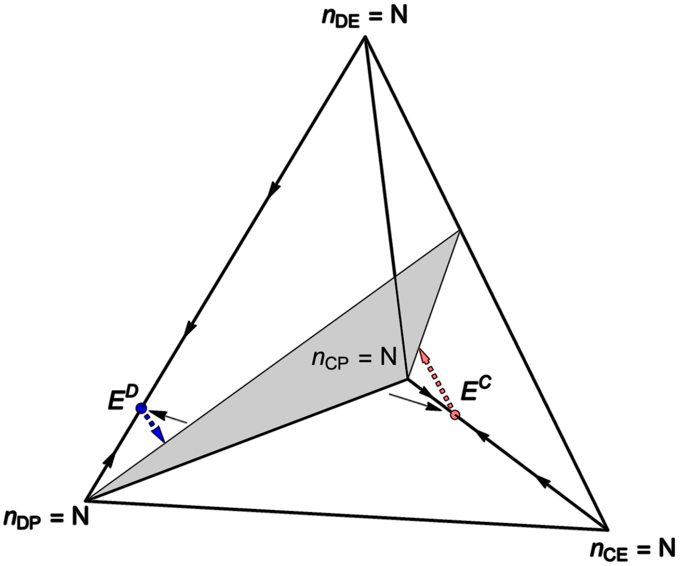

Fig. S1. The unperturbed best-response dynamic admits two equilibria (rest points of the dynamic): a cooperation equilibrium E^C in which only CP and CE are present, and a defection equilibrium E^D in which only DP and DE are present. Both equilibria are locally stable and their basins of attraction exhaust the state space. The basins of attraction are separated by the grey plane. Black arrows represent the flow of the dynamic. Stochastic stability analysis compares the relative stability of the two equilibria by comparing the number of revisions mistakes required to move between basins of attraction. Escape paths from the cooperation equilibrium and the defection equilibrium are represented by pink and blue arrows respectively.

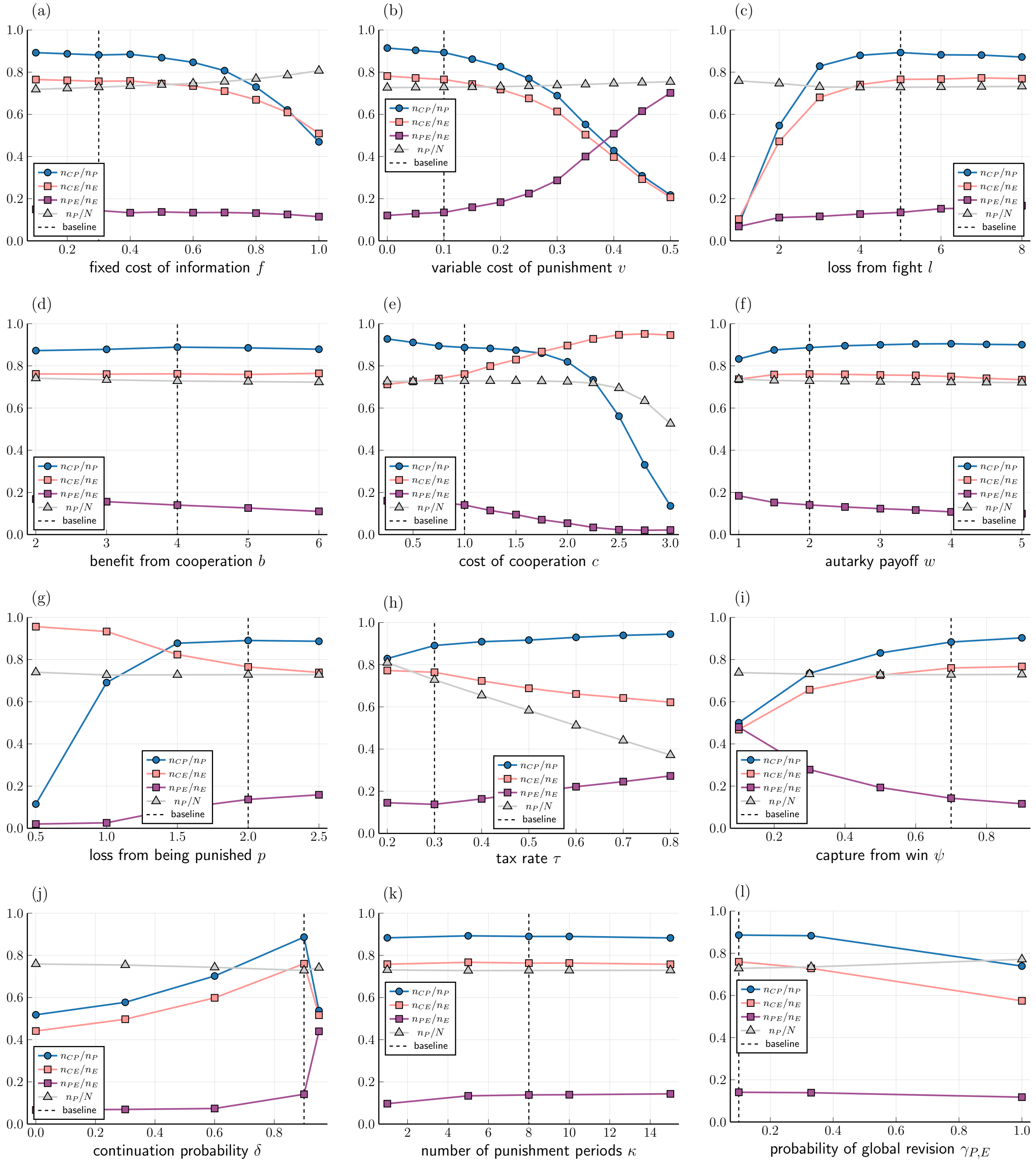

Fig. S2. The time averages of n_{CP}/n_P (circles), n_{CE}/n_E (light-coloured squares), n_{PE}/n_E (dark-coloured squares), and n_P/N (triangles) for different values of parameters. Dotted vertical lines indicate baseline values. **a** fixed cost f of information, **b** variable cost v of punishment, **c** loss l from being attacked, **d** benefit b of cooperation, **e** cost c of cooperation, **f** autarky payoff w, **g** loss from being punished p, **h** tax rate τ, **i** gain to unilateral attacker ψ, **j** continuation probability δ, **k** length of punishment κ, **l** revision probabilities γ; we compare the baseline model, in which the vector of revision probabilities is $(\gamma_{P,E}, \gamma_E, \gamma_P) = (0.1, 0.3, 0, 6)$, to the case of equally likely revision opportunities $(1/3, 1/3, 1/3)$, and the case where all revision opportunities are maximally permissive $(1, 0, 0)$.

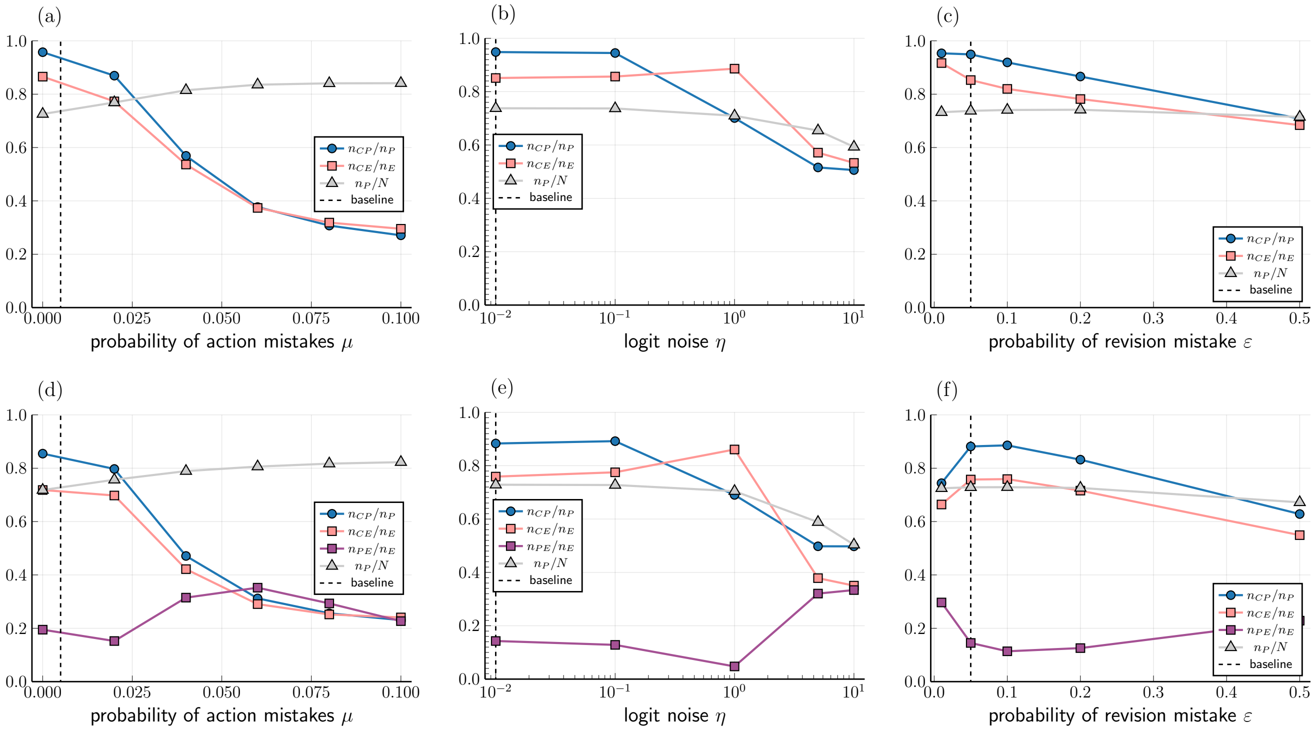

Fig. S3. The time averages of n_{CP}/n_P (circles), n_{CE}/n_E (squares) and n_P/N (triangles) for different values of **(a,d)** mistake probability μ, **(b,e)** logit precision η, **(c,f)** revision mistakes ε, when the set of enforcer strategies consists of CE and DE (a-c) or CE, DE, and PE (d-f). Dotted vertical lines indicate baseline values.

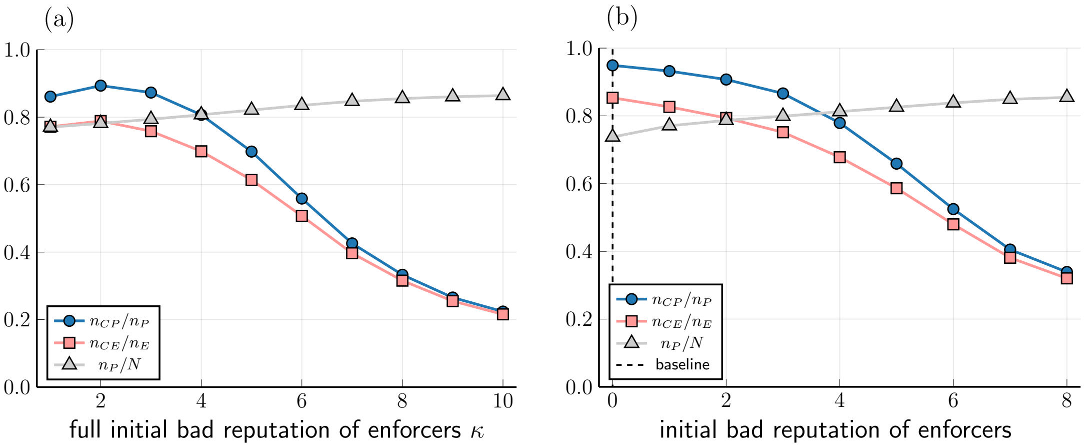

Fig. S4. The time averages of n_{CP}/n_P (circles), n_{CE}/n_E (squares) and n_P/N (triangles) for different values of parameters. **(a)** All enforcers begin with bad standing equal to κ (the number of punishment rounds), while κ varies from 1 to 10. **(b)** The number of punishment rounds κ is 8, as in the baseline. All enforcers begin with the same degree of bad standing, which varies from 0 (as in the baseline) to 8. All other parameters are the same as in our baseline in both panels.

Table S1. Model parameters for simulations: baseline and alternative values. Values not mentioned for the alternative are the same as in the baseline.

Parameter	Description	Baseline	Alternative
**General**			
δ	continuation probability	0.9	
κ	punishment rounds	8	
**Step 1: production**			
b	benefit	4	3
c	cost	1	2
w	autarky payoff	2	
**Step 2: enforcement**			
τ	tax rate	0.3	
v	variable cost	0.1	0.3
p	punishment	2	
f	fixed cost	0.3	0.4
**Step 3: meta-enforcement**			
ψ	capture from win	0.7	
l	loss from being attacked	5	4
**Revision and choice**			
μ	action mistakes	0.005	
η	logit imprecision	0.01	
ε	revision mistakes	0.05	
$\gamma_{P,E}$	global revision probability	0.1	
γ_E	enforcer revision probability	0.3	
γ_P	producer revision probability	0.6	

References

1. Kandori M (1992) Social norms and community enforcement. *The Review of Economic Studies* 59(1):63–80.
2. Ellison G (1993) Learning, local interaction, and coordination. *Econometrica* 61(5):1047–1071.
3. Blume LE (1993) The statistical mechanics of strategic interaction. *Games and economic behavior* 5(3):387–424.
4. Boyd R, Gintis H, Bowles S, Richerson PJ (2003) The evolution of altruistic punishment. *Proceedings of the National Academy of Sciences* 100(6):3531–3535.
5. Traulsen A, Nowak MA, Pacheco JM (2006) Stochastic dynamics of invasion and fixation. *Physical Review E* 74(1):011909.
6. Fudenberg D, Imhof LA (2006) Imitation processes with small mutations. *Journal of Economic Theory* 131(1):251–262.
7. Tarnita CE, Antal T, Nowak MA (2009) Mutation–selection equilibrium in games with mixed strategies. *Journal of theoretical biology* 261(1):50–57.
8. Antal T, Traulsen A, Ohtsuki H, Tarnita CE, Nowak MA (2009) Mutation-selection equilibrium in games with multiple strategies. *Journal of theoretical biology* 258(4):614–622.
